# Swarm-inspired magnetic metal-organic framework-based microbots for on-demand moisture manipulation

**DOI:** 10.1126/sciadv.aeg6193

**Published:** 2026-11-13

**Authors:** Mingren Cheng, Chenyang Tuo, Yifan Liang, Xinsheng Wang, Moyuan Cao, Xian-He Bu

**Affiliations:** ^1^ School of Materials Science and Engineering, Frontiers Science Center for New Organic Matter, Academy for Advanced Interdisciplinary Studies, Nankai University, Tianjin 300350, P.R. China.; ^2^ State Key Laboratory of Elemento-Organic Chemistry, College of Chemistry, Nankai University, Tianjin 300071, P.R. China.

## Abstract

Hygroscopic metal-organic frameworks (MOFs) are promising candidates for atmospheric water harvesting (AWH) and humidity control. Nevertheless, their practical application is often hindered by the poor manipulability of inherent powder forms and mass transfer limitations caused by the shaping. Drawing inspiration from the “hot defensive ball” aggregation morphology of honeybee swarms, a versatile magnetic shaping strategy hereby was reported to assemble MOF-based microbots, which enhances the formability of MOF powders. The obtained magnetic hygroscopic MOF (mMOF-303) exhibits enhanced water adsorption kinetics and enables efficient regeneration via multimodal heating strategies, including photothermal, electrothermal, and magnetothermal heating. Besides, the magnetic MOF-based microbots can change their aggregation patterns and perform directional motion according to application scenarios, enabling rapid water vapor sorption-desorption rates and precision confined space humidity control. This work provides a universal strategy to impart versatile formability to static MOF powders, paving the way for MOF engineering and advanced moisture management.

## INTRODUCTION

As a prominent class of porous coordination polymers, metal-organic frameworks (MOFs) possess ultra-high porosity, diverse pore architectures, and exceptional chemical and structural tunability, which have attracted significant attention across various fields (*1*–*4*). Hygroscopic MOFs, in particular, have emerged as state-of-the-art candidates for advanced moisture management (*5*–*7*). Compared with traditional sorbents such as silica gel and zeolites, MOF-based water sorbents demonstrate a superior performance combination (*8*–*10*). They exhibit high adsorption capacity over a wide range of relative humidity (RH) and steep uptake at low RH. Benefitting from mild regeneration temperatures, they show low energy requirements and enable the utilization of low-grade energy sources such as solar energy. The above features drive the exploration of hygroscopic MOFs for critical applications at the water-energy nexus, such as sorbent-assisted atmospheric water harvesting (SAWH), confined space humidity control, and adsorption-driven thermal management (*11*–*15*).

However, the practical application of MOFs is severely restricted by their as-synthesized microcrystalline powder form (*16*, *17*). On the one hand, powdery MOFs suffer from poor handling and mechanical instability, which renders them challenging to integrate into diverse functional devices. On the other hand, the unavoidable agglomeration of powders during operation creates a significant “stacking effect”, shielding moisture from accessing MOF particles and significantly impeding the macroscopic mass-transfer kinetics. As a result, the actual performance of MOF powders in practical systems often lags behind their theoretical limits.

To bridge the gap between microscopic MOF powders and macroscopic functional devices, various shaping strategies have been developed to construct MOF-based sorbents with diverse dimensions spanning from 0D to 3D (*18*–*21*). For instance, we recently developed an interfacial assembly technology to fabricate MOF-based silks with an ultrahigh MOF content exceeding 80 wt.% (*22*). When applied to SAWH, these silks exhibited a water adsorption rate three times faster than that of stacked MOF powders at the gram scale. MOF powders can also be mechanically compressed into thin sheets or freeze-dried into 3D porous monoliths, enabling rapid water adsorption kinetics and convenient integration into functional devices (*23*, *24*). Unfortunately, most conventional shaping methods typically require high pressure and/or binders (Fig. 1A). This is a trade-off between mechanical stability and high water adsorption performance (*25*). The high pressure used in compression will damage the fragile MOF framework, causing a permanent loss of porosity. Likewise, binders introduce extra weight and tend to coat the MOF particles. This blockage of active pores significantly increases the resistance to water transfer. In addition, these methods usually confine MOFs into fixed and static forms, making it difficult to adjust the structure for specific needs and hence limiting their practical versatility (*26*, *27*). Therefore, there is an urgent need to develop a simple and versatile shaping strategy that can reduce the reliance on high pressure and binders and at the same time facilitate flexible morphological reconfiguration based on application scenarios for superior water adsorption and high functional integration.

**Fig. 1.**
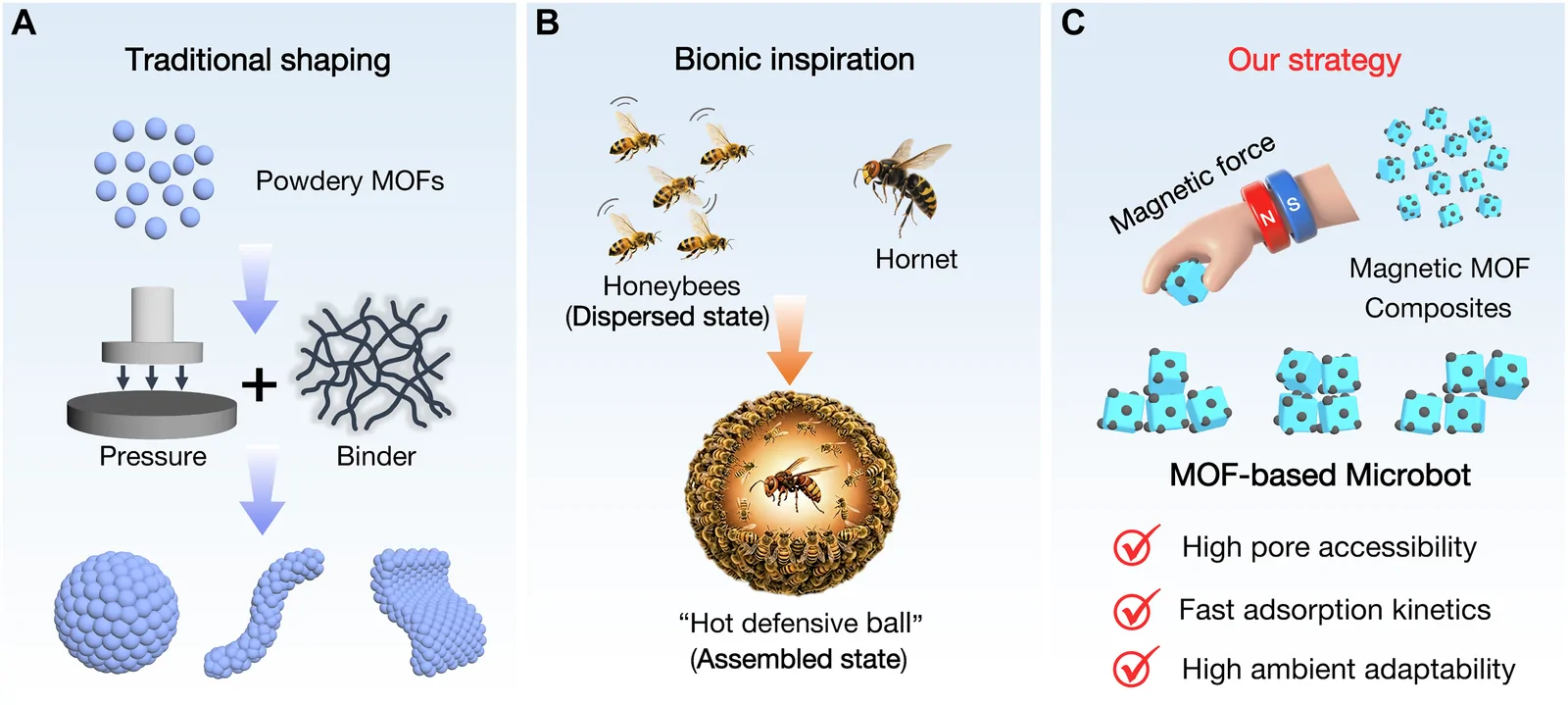
Schematic comparison between traditional shaping of MOFs and the proposed swarm morphology-inspired magnetic shaping strategy. (**A**) Traditional shaping methods relying on high pressure and/or binders, typically resulting in fixed, static forms. (**B**) Morphological inspiration derived from honeybees gathering into a “hot defensive ball” for collective defense. (**C**) The proposed magnetic shaping strategy fabricating MOF-based microbots and their associated advantages. Figures were created with Autodesk 3ds Max.

In nature, plants and animals always offer elegant inspiration for optimizing aggregation structures through collective behavior (*28*, *29*). For example, honeybees can use swarm behavior to defend against predatory hornets by aggregating into a compact “hot defensive ball” (Fig. 1B) (*30*). Inspired by the assembly morphology of natural creatures, here we propose a magnetic shaping strategy to overcome the intrinsic processing limitations of MOF water sorbents. By integrating hygroscopic MOFs with magnetic nanoparticles, we fabricated magnetic MOF composites (termed MOF-based microbots) that execute field-driven morphological reconfiguration and directional motion (*31*–*33*), exhibiting several unique advantages (Fig. 1C). First, the pressure- and binder-free shaping retains the high pore accessibility from the original MOFs. The magnetic MOF composite (mMOF-303) with 80.9 wt % MOF-303 content exhibits a high water adsorption capacity of 0.34 g g^−1^ at 30% RH. Second, the MOF-based microbot can adhere to various magnetic substrates for atmospheric water capture and achieve rapid adsorbed water release via multimodal heating approaches, such as photothermal, electrothermal, and magnetothermal heating. Upon fluidization by a convective airflow, the mMOF-303 exhibits an exceptional water sorption rate of up to 17.8 mg g^−1^ min^−1^ under a low-humidity regime (30% RH). Third, the hygroscopic MOF-based microbots leverage directional mobility for targeted moisture management, enabling them to adsorb water in high-RH environments and release it in low-RH environments. Such dynamic responsiveness ensures reliable, broad-range humidity mitigation (15–85%) within confined environments, demonstrating an exceptional capacity for precision on-demand moisture management. This work presents a versatile and smart strategy to transform intractable MOF powders into functional applications, providing significant promise for the rational engineering of MOFs in advanced moisture manipulation.

## RESULTS

### Properties of mMOF-303 microbots

To implement the swarm-inspired magnetic shaping strategy, a facile in situ growth approach was used to assemble magnetic hygroscopic MOF-based microbots, mMOF-303 (Fig. 2A), in which polydopamine (PDA) coated Fe_3_O_4_ (Fe_3_O_4_@PDA) was employed as the magnetic core. As shown in the FTIR spectra (fig. S1), the characteristic peaks at 3423 cm^−1^ and 1623 cm^−1^ of Fe_3_O_4_ were assigned to the stretching and bending vibrations of hydroxyl groups, respectively, while the peak at 567 cm^−1^ is attributed to the Fe-O vibration. After surface modification, the emerging peaks at 1488 cm^−1^ and 1284 cm^−1^ originate from the skeletal vibrations of C=C (aromatic ring) and amine groups of PDA (*34*, *35*). The abundant catechol and amine groups of the PDA layer effectively coordinate with Al^3+^ ions, serving as nucleation sites to induce the in situ growth of MOF-303. Benefiting from its excellent magnetic responsiveness, the as-synthesized mMOF-303 can be conveniently isolated and purified from the mother liquor via magnetic separation (Fig. 2B). The final sample was obtained as a brown powder after the drying process and the color of powder gradually deepens with the increasing Fe_3_O_4_@PDA loading (mMOF-303-2, mMOF-303-3 and mMOF-303-4, fig. S2). Morphological characterization reveals that the Fe_3_O_4_ nanoparticles are uniformly dispersed with an average diameter of less than 20 nm. In addition, a thin PDA layer is observed coating the surface of the Fe_3_O_4_ nanoparticles, confirming the successful surface modification (figs. S3 and S4). As shown in Fig. 2, C and D, the mMOF-303 exhibits a characteristic nanoparticle morphology, in which the MOF-303 is integrated with the Fe_3_O_4_@PDA magnetic core. It is worth noting that to balance the reaction throughput and processing speed for scalable fabrication, a rapid synthesis route was adopted, which naturally introduces minor morphological variations and size heterogeneity among the composite crystals, as is also intrinsically observed in pristine MOF-303 (fig. S5). Corresponding energy dispersive x-ray spectroscopy (EDS) mapping images reveal the homogeneous distribution of Fe, Al, N and O elements (Fig. 2E), further confirming the successful construction of the MOF-based microbots. Similarly, such consistent elemental distribution and integrated morphology are equally preserved in the microbots with higher Fe_3_O_4_@PDA contents (fig. S6). Consequently, these minor microscale morphological variations do not adversely affect the magnetic interactions. The structural integration guarantees effective control and versatility for subsequent magnetic-driven applications (*36*).

**Fig. 2.**
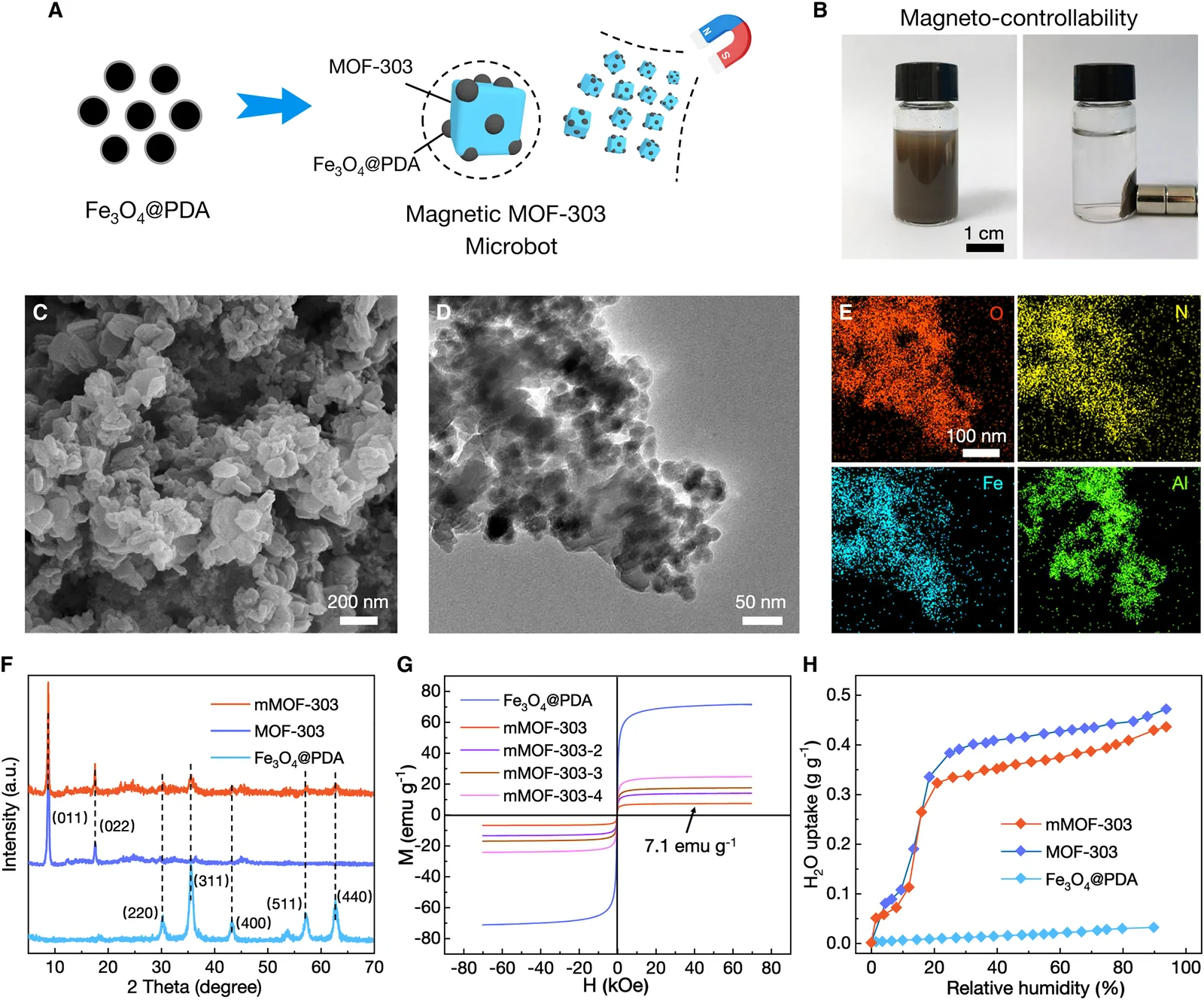
Preparation and characterization of magnetic MOF-303 composites (mMOF-303). (**A**) Schematic diagram of the preparation process of mMOF-303. The illustration was created with Autodesk 3ds Max. (**B**) Photographs showing excellent magnetic responsiveness of the prepared mMOF-303. (**C**) SEM image, (**D**) TEM image and (**E**) corresponding elemental mapping images of mMOF-303. (**F**) PXRD patterns of Fe_3_O_4_@PDA, MOF-303 and mMOF-303. (**G**) Magnetic hysteresis loops of Fe_3_O_4_@PDA, mMOF-303, mMOF-303-2, mMOF-303-3 and mMOF-303-4. (**H**) Dynamic water adsorption isotherms of Fe_3_O_4_@PDA, MOF-303 and mMOF-303.

Additionally, the crystalline structures of the mMOF-303 with different MOF-303 contents were verified by powder x-ray diffraction (PXRD) (Fig. 2F and fig. S7). The characteristic diffraction peaks at 2θ = 8.7° and 17.5° are assigned to the (011) and (022) crystal planes of MOF-303, respectively (*37*). Meanwhile, the additional diffraction signals located at 30.4°, 35.7°, 43.3°, 57.3°, and 62.8° are attributed to the (220), (311), (400), (511), and (440) planes of Fe_3_O_4_ (*38*). These results indicate that the in situ hybridization process yielded MOF-based microbots with high crystallinity and structural integrity. Importantly, this binder-free strategy yields an ultrahigh MOF loading. Thermogravimetric analysis (TGA) results reveal that mMOF-303 possesses a high MOF mass content of 80.9 wt.% (fig. S8), which is higher than that of many conventional MOF shaping methods employing high pressure and/or inactive binders. As a result, mMOF-303 exhibits a high Brunauer-Emmett-Teller (BET) specific surface area (1178.8 m^2^ g^−1^) (fig. S9). This demonstrates that it has a robust structural foundation with high intrinsic pore accessibility for the subsequent moisture capture. The magnetic properties of the magnetic microbots were also investigated using a vibrating sample magnetometer (VSM) (Fig. 2G). The magnetization curves of both Fe_3_O_4_@PDA and other MOF-based magnetic samples pass through the origin and show very low remanence and coercivity, displaying typical superparamagnetic behavior (*39*). Notably, although the saturation magnetization (*M_s_*) of mMOF-303 decreases obviously compared to Fe_3_O_4_@PDA due to the high MOF loading, it still can achieve 7.1 emu g^−1^.

To evaluate the moisture capture performance, water vapor sorption isotherms and adsorption kinetics were investigated. As shown in fig. S10, consistent with pristine MOF-303, MOF-based magnetic samples exhibit steep S-shaped water adsorption isotherms attributed to the cooperative filling of their hydrophilic pores. Importantly, despite the increasing *M_s_* with higher magnetic loadings, all variants retain this characteristic S-shaped profile without significant shifts in the critical inflection point, confirming that the magnetic loading can be flexibly tailored for application scenarios requiring higher magnetic responsiveness. A comprehensive comparison of magnetic properties and water adsorption capabilities across all four variants is summarized in table S1. Based on these results, the mMOF-303 was chosen as the primary representative sample for subsequent studies, as it prioritizes and maximizes the moisture adsorption capacity while satisfying the requirements for static magnetic adhesion and dynamic magnetic actuation. Dynamic water vapor sorption isotherms also confirm that mMOF-303 maintains water adsorption characteristics similar to those of pristine MOF-303 (Fig. 2H). Specifically, mMOF-303 exhibits a prominent adsorption inflection point located below 15% RH and achieves a competitive water uptake capacity of 0.34 g g^−1^ at 30% RH, which is highly advantageous for water vapor adsorption in arid environments (*40*). Because the water uptake kinetics are predominantly governed by the intrinsic micropore accessibility and intra-crystalline diffusion within the MOF framework, the microscale crystal size differences do not alter the transport resistance inside the pores. Moreover, the dynamic water uptake profiles (fig. S11) show that mMOF-303 reaches rapid saturation across a wide range of humidity levels (20–50% RH) and as the RH increases, both the equilibrium adsorption capacity and the kinetic adsorption rate exhibit an enhancement. Such rapid moisture adsorption kinetics further highlights the superiority of the binder-free assembly strategy, as it eliminates the risk of pore clogging and active-site shielding in conventional shaping. Consequently, mMOF-303 combines high adsorption capacity and fast kinetics, making it a promising candidate for advanced moisture management. To further evaluate the stability of mMOF-303 during adsorption-desorption cycling, multiple cycles were performed (fig. S12). After 20 cycles, mMOF-303 exhibits no degradation in its water adsorption capacity, while maintaining an adsorption rate comparable to that of the pristine sample. In addition, both the characteristic XRD peaks and the saturation magnetization of mMOF-303 exhibited no significant changes after cycles (figs. S13 and S14). All these results strongly demonstrate the outstanding stability of mMOF-303 during the adsorption-desorption cycling process.

### Static and dynamic magnetic shaping

Beyond the exceptional moisture capturing capacity, the outstanding feature of the mMOF-303 microbot is its superior magnetic manipulability and versatile environmental adaptability (*41*). Traditional SAWH systems are generally shaping hygroscopic MOFs into fixed and monolithic forms such as granules, pellets or films, lacking the capacity for flexible morphological switching. Such rigid configurations often limit the efficiency of moisture capture in non-planar or spatially constrained environments. Conversely, mMOF-303 microbots can serve as a reconfigurable and mobile sorbent platform, allowing for versatile shape-switching to meet various moisture manipulation needs.

The versatility of mMOF-303 structure is first shown by its ability to on-demand assembly onto various magnetic substrates (Fig. 3A). In order to explore this capability, we made several magnetic templates with various geometries as shown in fig. S15. In the presence of such magnetic templates, mMOF-303 microbots get captured and patterned almost instantaneously, replicating the complex geometries of the underlying magnetic field (Fig. 3B). This rapid response can be made to adapt to any given situation in a flexible, demand-driven way as opposed to traditional shaping which is very rigid. To further evaluate the ambient versatility, mMOF-303 was integrated with portable magnetic adhesive tape which can be easily attached to object surfaces (fig. S16). Also, the adhesion system can be designed to have a reversible state switching if external magnetic fields are applied and taken away. As can be seen in Fig. 3C, the mMOF-303 microbots can switch between a fixed adhering state and the original loose powder state. This is important for the efficient recovery and subsequent transport of the sorbent after moisture capture. In addition to morphological flexibility, they also have mechanical stability. For example, compared with pristine loose MOF-303 powder, the mMOF-303 exhibits improved wind resistance with the effect of the magnetic field (figs. S17 and S18). When airflow speed is high at 5 m s^−1^, magnetically fixed mMOF-303 maintains its structural integrity and does not get dispersed, but the loose powder gets lost to the environment. Even under a continuous airflow of up to 10 m s^−1^ for 10 seconds, only approximately 10% of mMOF-303 powder was dispersed, proving the high binding strength. The enhanced stability ensures that the moisture manipulation system can operate reliably in harsh or outdoor environments, effectively bridging the gap between laboratory research and real-world applications.

**Fig. 3.**
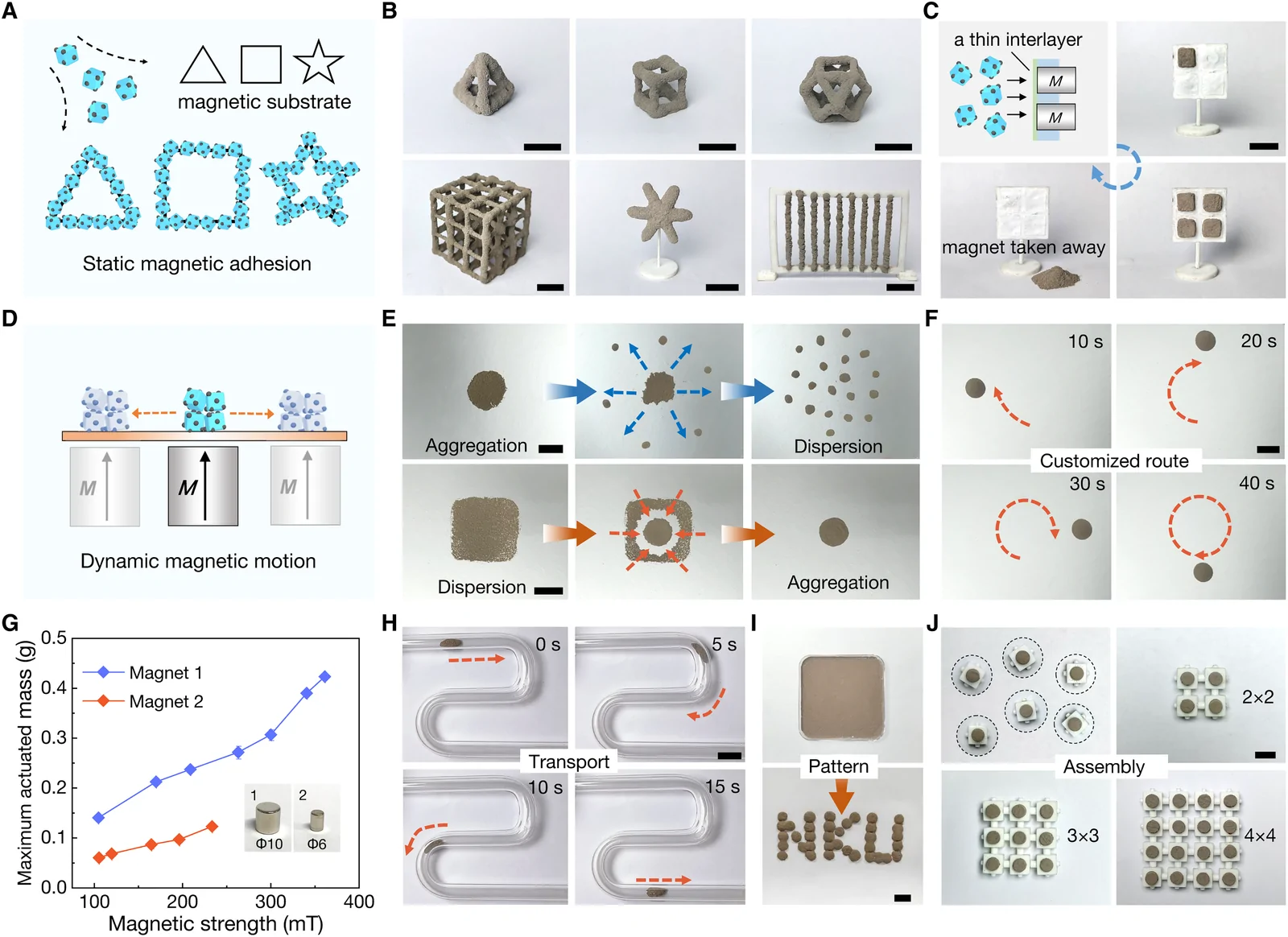
Static shaping and dynamic manipulation of MOF-based magnetic microbots. (**A**) Schematic illustration of static magnetic adhesion onto various shaped substrates. The illustration was created with Autodesk 3ds Max. (**B**) Photographs demonstrating the assembly of mMOF-303 microbots onto diverse magnetic substrates. (**C**) Reversible transition between a magnetically adhered shaped state and a loose powder state upon application and removal of the magnetic field with a magnetic field strength of ∼500.9 mT. The illustration was created with Autodesk 3ds Max. (**D**) Schematic illustration of dynamic motion driven by a moving magnetic field. The illustration was created with Autodesk 3ds Max. (**E**) Regulation of microbot collective states, showing the transitions between aggregated and dispersed state, and vice versa driven by magnetic actuation. (**F**) Photographs showing a small pile of microbots moving along a customized circular route. The applied magnetic field strength is ∼383.8 mT and the magnetic actuation speed is approximately 2.6 mm s^−1^. (**G**) Maximum actuated mass of mMOF-303 powders as a function of surface magnetic field strength. (**H**) Directional motion of mMOF-303 microbots through a sealed and complex glass channel. The applied magnetic field strength is ∼318.2 mT and the magnetic actuation speed is approximately 3.2 mm s^−1^. (**I**) Programmable patterning of mMOF-303 powder into specific letters. (**J**) Modular assembly of mMOF-303-coated magnetic units into larger arrays. All scale bars are 1 cm.

In addition to static shaping, the dynamic magnetic actuation of mMOF-303 microbots was also explored (Fig. 3D). Although basic switching was previously demonstrated in Fig. 3, C and E, and corresponding videos (movies S1 and S2) highlight a higher degree of flexibility in dynamic transitioning between dispersed and aggregated states by external magnetic actuation. This rapid reconfiguration allows the microbot swarm to fit its quantity and shape in real-time. Using programmed magnetic sequences, the mMOF-303 swarm can also move along customized routes like a circle (Fig. 3F and movie S3). We further quantitatively analyzed the relationship between the surface magnetic field strength and the dynamic behavior of mMOF-303 during the actuation process. The mechanism of magnetic actuation lies in utilizing the non-uniform magnetic field generated by a moving permanent magnet to exert an oblique downward macroscopic Kelvin gradient force on the powder, whose horizontal component overcomes substrate friction to provide translational driving power, while its vertical component produces a magnetic clamping effect (fig. S19). As shown in Fig. 3G, the maximum actuated mass of mMOF-303 exhibits a monotonic, non-linear increase with the enhancement of the magnetic field strength. Therefore, the mMOF-303 microbots also adapt to more confined environments, such as complex and sealed pipes (Fig. 3H and movie S4) where fixed stationary sorbents are limited by their rigidity. They can pass directionally through such limited space, which is beneficial for MOF-based sorbent to carry convenient local moisture manipulation on the specific spots. The system’s versatility is also seen in personalized patterning (Fig. 3I), where the mMOF-303 microbot is molded into unique functional geometries via specific magnetic templates (fig. S20). Notably, the magnetically molded patterns can remain intact in a vertical orientation for over 1 week, demonstrating robust structural stability (fig. S21). Moreover, we have created a modular assembly to fit the demand for a large amount. The individual magnetic units incorporated with mMOF-303 can also be integrated into a large array (Fig. 3J and fig. S22) such that the microbot system can be scaled up or reconfigured on demand. In contrast, conventional compression molding can only shape MOF-303 powder into rigid pellets (fig. S23), which are highly susceptible to fragmentation during the molding and subsequent handling processes, making them difficult to adapt to diverse application requirements. The demonstrated capabilities of mMOF-303 suggest a useful way to overcome the spatial limitations of stationary systems, showing the practical utility for versatile real-world moisture manipulation.

### Moisture adsorption and desorption

Building on the magnetic adhesion and dynamic control of mMOF-303, we have also taken into account the moisture adsorption and desorption performance to advance its potential for practical applications. In general, the moisture adsorption performance of powdery MOFs is limited by the stacking effect, which leads to a high internal mass transfer barrier (*25*). Furthermore, the structural hurdle is also caused by slow natural convection in ambient air, severely impeding the external diffusion of moisture towards internal adsorption sites (*42*, *43*). To deal with this, we leveraged the magnetic adhesion of mMOF-303 and enhanced wind resistance to construct a strong adsorption module to evaluate the adsorption kinetics of both static and airflow-assisted situations (Fig. 4A). MOF-303 powder with 50 mg cm^−2^ loading density under 30% RH static condition requires more than 400 minutes to reach adsorption equilibrium. In contrast, with the introduction of an airflow (Fig. 4B), the saturation rate is effectively doubled, and the mMOF-303 exhibits a high water uptake capacity of 0.31 g g^−1^. The remarkable acceleration in moisture capture kinetics indicates that an external airflow can greatly enhance the efficiency of gas-solid contact. This optimization is directly enabled by our pressure-free magnetic shaping strategy, which maintains a highly porous and loosely aggregated configuration that allows the convective airflow to thoroughly penetrate the powder swarm without encountering high flow resistance. And also, higher moisture adsorption rate is observed as the mMOF-303 loading drops (Fig. 4C), indicating the significant impact of the stacking effect. In parallel to the external airflow assistance, we also explored an active convection method using the integration of mMOF-303 on a rotating magnetic platform (fig. S24 and movie S5) for mechanical convection enhancement. It was found that the high relative velocity between mMOF-303 and the surrounding air greatly speeds up the rate of moisture adsorption (fig. S25). Such an active design provides another effective strategy to reduce the diffusion limitations, providing much faster moisture capture kinetics via mechanical actuation.

**Fig. 4.**
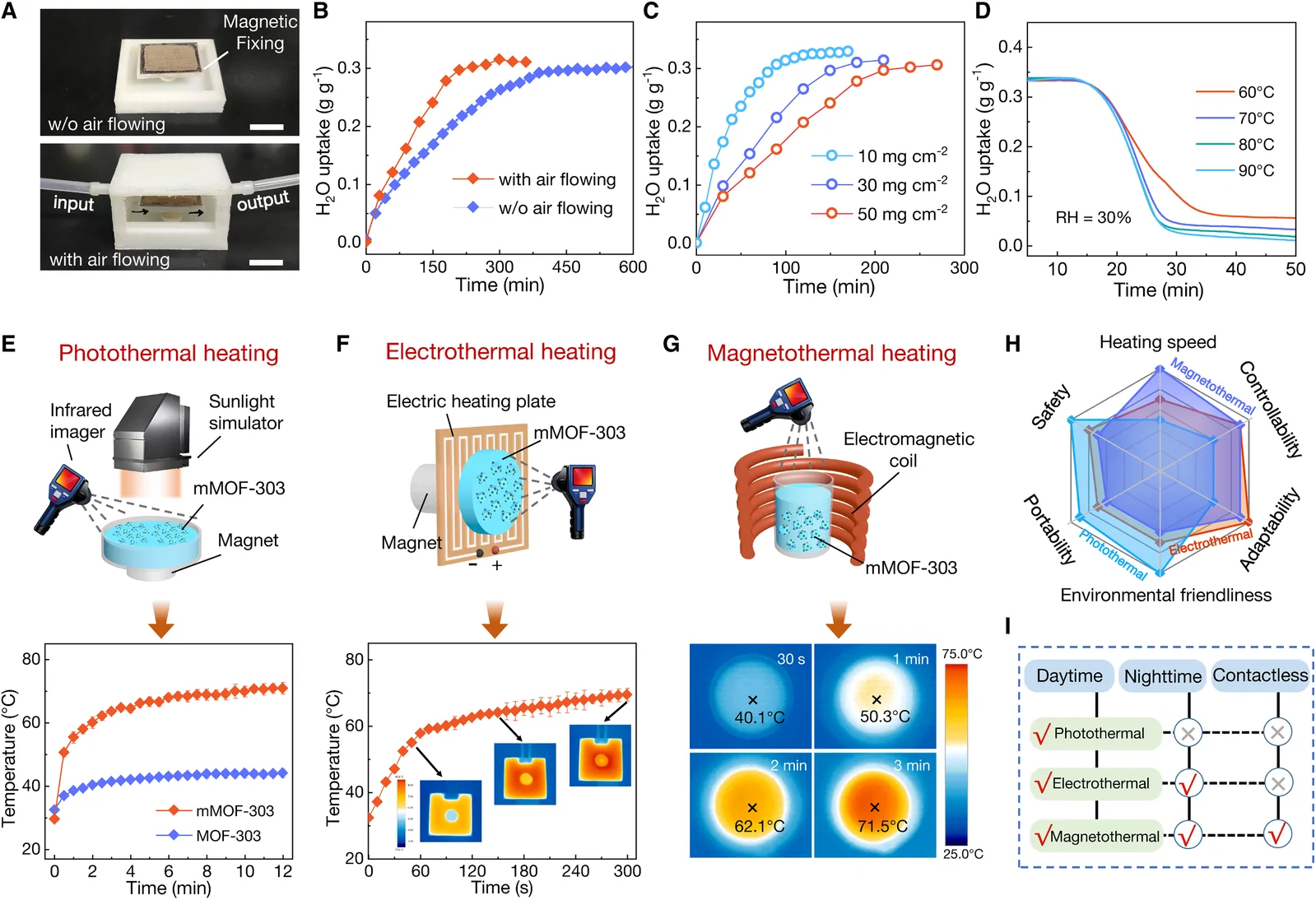
Water adsorption and multimodal heating-assisted desorption of mMOF-303. (**A**) Photographs of the testing setup for water adsorption of mMOF-303 with and without air flowing. Scale bars, 2 cm. (**B**) Dynamic water uptake curves of mMOF-303 with and without air flowing at 30% RH. Sample loading density is 50 mg cm^−2^. (**C**) Dynamic water uptake curves of mMOF-303 with different loadings at 30% RH. (**D**) Water desorption curves of mMOF-303 at different temperatures and 30% RH. (**E**) Schematic illustration of photothermal heating measurement for mMOF-303 (up) and corresponding time-dependent temperature change curves of MOF-303 and mMOF-303 (down). (**F**) Schematic illustration of electrothermal heating measurement for mMOF-303 (up) and corresponding time-dependent temperature change curve (down). Sample loading density is 50 mg cm^−2^. Inset: the corresponding infrared thermal images. (**G**) Schematic illustration of magnetothermal heating measurement for mMOF-303 (up) and corresponding infrared thermal images at different durations (down). The illustrations were created with Autodesk 3ds Max. (**H**) Radar illustration comparing three heating methods. (**I**) Schematic illustration of applicability of different heating methods in various conditions.

As well as moisture adsorption capacity, the sustainability of an atmospheric moisture capturing cycle relies on fast and efficient release of water for sorbent regeneration with minimal energy input (*44*, *45*). Encouragingly, water-adsorbed mMOF-303 is highly responsive to moderate thermal stimuli. As shown in Fig. 4D, when the temperature reaches a threshold of approximately 70°C, nearly 88.3% of the adsorbed moisture can be released within 35 minutes. This moderate temperature regeneration capability enables excellent compatibility with diverse heating strategies and thermal sources. Consequently, we evaluated the feasibility of diverse heating approaches for mMOF-303 microbots, including photothermal, electrothermal and magnetothermal heating. As illustrated by the UV-vis-NIR absorption spectra in fig. S26, mMOF-303 displays enhanced light absorbance across the range from 300 to 2500 nm compared with the pristine MOF-303, which can be attributed to the high photothermal conversion capability of the PDA layer. Therefore, under 1.0 sun illumination, the surface temperature of mMOF-303 powder rapidly increases to over 70°C within 10 minutes (Fig. 4E and fig. S27). Furthermore, photothermal performance across all variants confirms that their solar-driven heating capabilities are progressively enhanced with increasing Fe_3_O_4_@PDA loading (fig. S28), ensuring robust thermal support for photothermal desorption. In the absence of solar irradiation, electrothermal heating serves as a precisely tunable alternative (*46*). By controlling the applied power of the heating plate, mMOF-303 can similarly reach the required desorption temperature within a few minutes (Fig. 4F). Moreover, the magnetic adhesion enables mMOF-303 to remain fixed to the heating plate regardless of orientation. This allows for operation in hanging positions (fig. S29) and expands its practical application scenarios. Notably, we can see the decreasing amount of the time required at a lower areal density (fig. S30). It is due to the relatively low thermal conductivity of mMOF-303 (0.101 W m^−1^ K^−1^, fig. S31). Unlike photothermal and electrothermal approaches, the magnetothermal strategy gets rid of the thermal conductivity limitation (*47*). Driven by a customized high frequency alternating magnetic field (fig. S32), the mMOF-303 microbots can act as localized internal heat sources (Fig. 4G). By generating heat directly within the sorbent matrix via electromagnetic induction, the system reaches 71.5°C within only 3 minutes.

By comparing various dimensions of aspects heating speed, safety, controllability, portability, environmental adaptability and friendliness, it was revealed that the three heating methods provide different yet complementary advantages (Fig. 4H). Photothermal heating is a sustainable and green choice for use in the daytime, while electrothermal and magnetothermal modes bridge the gap when solar radiation is unavailable such as during nighttime. Moreover, the magnetothermal heating also has a special non-contact capability feature, which is convenient to perform efficient regeneration in special environments with physically inaccessible sorbents (Fig. 4I). The multimodal versatility of the mMOF-303 reveals that it is not merely a high-performance water sorbent, but also a superior platform capable of rapid moisture release.

### Efficient moisture capture and humidity control

In addition to the fundamental material characterizations, we also evaluated the practical applications of moisture manipulation of mMOF-303 microbots, including SAWH and confined space humidity regulation. For SAWH, the aforementioned air-assisted strategy can enhance convection between the air and the sorbent bulk. Improving the contact between individual particles and moisture within the stacked bulk is another effective approach. According to Fig. 5A and movie S6, we have investigated an air-driven fluidization strategy and also used magnetic control to collect the mMOF-303 microbots for repeated fluidization operations. By introducing an upward humidified gas jet into the sieved powder with particle sizes under 10 μm at an outlet velocity of 6.9 m s^−1^, the mMOF-303 microbots transition from a static packed state into a highly dispersed and suspended state. This dynamic jet dispersion process induces intense relative particle gas motion, which substantially enhances the contact area between the moisture and the powder while compressing the local mass transfer boundary layer down to a few micrometers. As a result, the external gas film resistance to vapor transport is eliminated, allowing water molecules to rapidly reach the particle surface and fully liberating the intrinsic micropore diffusion kinetics of mMOF-303. At the same time, the mMOF-303 microbots are anchored by an external magnetic field to prevent material loss, leveraging their capacity for static magnetic shaping. To facilitate subsequent fluidization, the powders are directionally driven back to the bottom via magnetic actuation to complete the collection and reset for the next operation. This process integrates the intrinsic sorption capability of static frameworks with magnetic actuation, further demonstrating the superior utility of our magnetically configured MOF system. This dynamic behavior is much faster than static standing to accelerate moisture diffusion into the MOF particles. Therefore, at about 30% RH, the fluidized mMOF-303 microbots can quickly approach moisture saturation within only 18 minutes, and the adsorption rate is as high as 17.8 mg g^−1^ min^−1^, which is much higher than the standing sample (Fig. 5B). Although direct kinetic comparisons across different literature studies are inherently subject to variations in operating conditions, it is noteworthy that even under such a low RH regime, this high adsorption rate surpasses most values reported in recent literature (*22*–*24*, *48*–*52*), which shows the advantages of the mMOF-303 microbot dynamic shaping system (Fig. 5C and table S2).

**Fig. 5.**
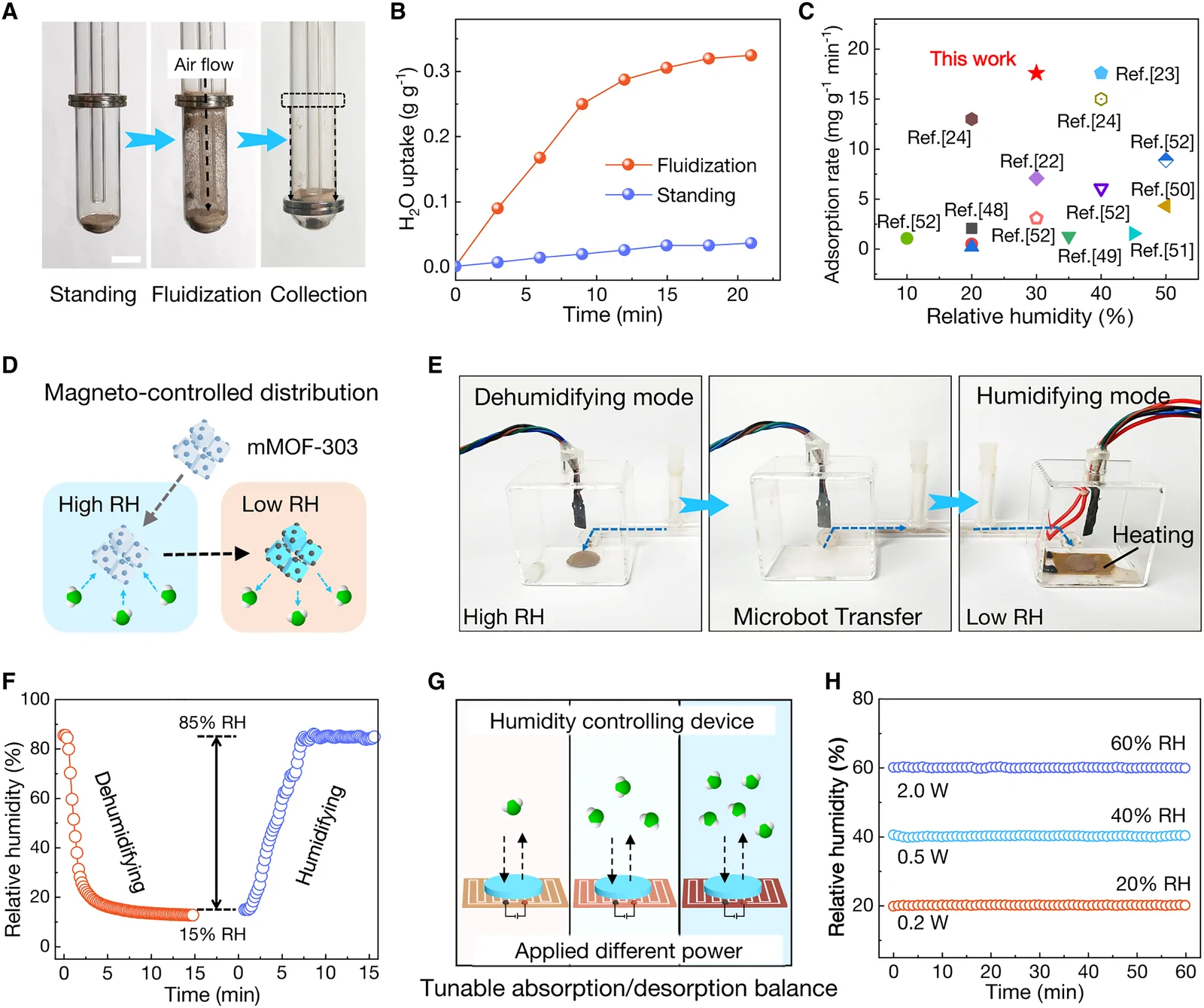
Moisture capture and humidity regulation via MOF-based microbots. (**A**) Photographs showing the fluidization process of mMOF-303 powder for enhanced water adsorption. (**B**) Dynamic water uptake curve comparison of mMOF-303 powder with standing and fluidization at 30% RH. (**C**) Comparison of water adsorption rate of fluidized mMOF-303 powder with representative MOF-based sorbents. (**D**) Schematic illustration and (**E**) device display of on-demand humidity regulation of MOF-based microbots via magnetically directional motion. The illustration was created with Autodesk 3ds Max. (**F**) Time-dependent humidity change curves in the process of dehumidifying and humidifying. (**G**) Schematic illustration of constant humidity regulation via power control of electric heating plate. The illustration was created with Autodesk 3ds Max. (**H**) Corresponding time-dependent humidity control curves by applying different powers.

Following the effective moisture adsorption, quantitative water desorption experiments of mMOF-303 were carried out to evaluate the release performance across different heating modes (fig. S33). Specifically, under simulated one-sun irradiation, mMOF-303 releases approximately 68.5% of the adsorbed water within 60 minutes. Meanwhile, over 77.2% of the adsorbed water can be released within 45 minutes. Magnetothermal actuation demonstrates higher water desorption efficiency, releasing approximately 92.9% of the adsorbed water within a mere 20 minutes. To better visualize the water adsorption and desorption process of mMOF-303 under three heating modalities, we performed in-situ monitoring of the mMOF-303 within a confined container. As shown in the figs. S34 to S36 and movies S7 to S9, the liquid water droplets gradually appeared during heating. Once the heat source was removed, the released moisture was reversibly re-adsorbed by mMOF-303. These characterizations successfully proved the efficacy of our magnetic shaping strategy and demonstrated that the resulting architecture enables rapid moisture release kinetics, establishing a solid material level foundation for mass transfer. It should be noted that the present work focuses on establishing the magnetic shaping method and proving its water capture and release feasibility at the sorbent level, rather than constructing a fully operational macroscale water harvesting device. Practical liquid water collection and overall efficiency in real world scenarios will further depend on downstream condensation units and system thermal management.

Besides moisture harvesting, the mMOF-303 microbots with magnetic responsiveness also enable active and on-demand humidity regulation. The microbots as mobile moisture carriers are shown in Fig. 5D. They were magnetically guided to specific functional zones, where they capture excess moisture or release stored vapor according to the humidity level. To evaluate the intelligent regulation under closed conditions, we set up a prototype chamber (Fig. 5E and fig. S37). With magnetic manipulation and precise thermal actuation, the microbots can switch rapidly between humidification and dehumidification modes over a broad range of 15% to 85% RH (Fig. 5F). Specifically, the regulation process can be completed within 10 minutes, regardless of whether it operates in humidification or dehumidification mode, which is also verified in the subsequent stepwise humidity tests (fig. S38). To further verify this durability, consecutive humidity regulation cycles were performed. As illustrated in fig. S39, the mMOF-303 microbots efficiently maintained their humidity regulation capability over the broad range throughout 5 cycles, highlighting their excellent performance stability. Furthermore, we explored a constant humidity regulation strategy via electrothermal power control (Fig. 5G), in which adsorption rates reach dynamic equilibrium with ambient humidity. As shown in Fig. 5H, the humidity control curves indicate that the system can maintain various target humidity levels over long-term durations, with humidity fluctuations not exceeding 0.5% RH. Consequently, the mMOF-303 microbots establish a versatile platform for targeted humidity management, surpassing the physical limitations of passive static materials and demonstrating the clear operational superiority of magnetic control in specialized confined spaces. Future developments focusing on intelligent, autonomous control will further bridge the gap between lab-scale prototypes and real-world implementation in larger, more complex environments.

## DISCUSSION

In summary, inspired by the collective morphology of honeybee swarms, we report a versatile magnetic shaping strategy transforming hygroscopic MOF powders into multifunctional microbots. The binder-free approach overcomes the traditional trade-off between structural formability and mass transfer kinetics and preserves the high porosity of MOF-303. Therefore, the mMOF-303 microbots under fluidization obtain a high sorption rate of 17.8 mg g^−1^ min^−1^. In addition, they can achieve rapid moisture release via versatile heating modes. The magnetic responsiveness for directional motion and morphological reconfiguration also enables active and on-demand humidity regulation across a wide range from 15% to 85% RH. Ultimately, this work provides a universal paradigm to endow hygroscopic MOF powder with on-demand mobility. This swarm-inspired strategy paves the way for shaping MOF-based sorbents and offers promising potential for advanced moisture manipulation.

## MATERIALS AND METHODS

### Chemicals and materials

3,5-Pyrazoledicarboxylic acid monohydrate (H_2_PZDC·H_2_O, 97.0%), Dopamine hydrochloride (DA·HCl, 98.0%), Poly(vinylpyrrolidone) (PVP-K25, 99.0%) and ammonia solution (28.0%–30.0%) were purchased from Shanghai Aladdin Biochemical Technology Co., Ltd. Aluminum chloride hexahydrate (AlCl_3_·6H_2_O, AR) was purchased from Tianjin Heowns Biochemical Technology Co., Ltd. Sodium hydroxide (NaOH, AR) was purchased from Tianjin Damao Chemical Reagent Factory. Tris(hydroxymethyl)aminomethane (Tris, 99.0%) was purchased from J&K Scientific. Iron (III) chloride hexahydrate (FeCl_3_·6H_2_O, 99.0%) and Iron (II) chloride tetrahydrate (FeCl_2_·4H_2_O, 99.0%) were purchased from Shanghai Macklin Biochemical Technology Co., Ltd. All chemicals and materials were used without further purification.

### Synthesis of Fe_3_O_4_ and Fe_3_O_4_@PDA

Magnetic Fe_3_O_4_ cores were synthesized according to the following procedure. Typically, FeCl_3_·6H_2_O (5.0 g) and FeCl_2_·4H_2_O (2.5 g) were dissolved in 100 ml of deionized (DI) water. The reaction was conducted at 90°C under a nitrogen environment with mechanical stirring. Subsequently, 25 ml of ammonia solution was added dropwise and the color of the mixture changed from dark yellow to black. After 30 minutes of continuous stirring and cooling to ambient temperature, the resulting black Fe_3_O_4_ precipitates were obtained via magnetic separation and repeated washing with DI water. Finally, the obtained Fe_3_O_4_ was dispersed in DI water for further use. To prepare Fe_3_O_4_@PDA, the aqueous dispersion containing 1.0 g of Fe_3_O_4_ was mixed with a 200 ml Tris buffer solution (pH = 8.5) containing 100 mg PVP-K25 via sonication. Subsequently, 10 ml DA·HCl (25 mg ml^−1^) solution was added to the suspension. The reaction proceeded overnight at room temperature. The prepared Fe_3_O_4_@PDA was isolated magnetically and washed with DI water for several times. Finally, the Fe_3_O_4_@PDA powder was obtained by drying under vacuum at 80°C for 12 hours.

### Synthesis of MOF-303 and mMOF-303 variants

The MOF-303 was synthesized according to the following procedure. Specifically, 1.74 g of H_2_PZDC·H_2_O and 1.2 g NaOH were dissolved in 50 ml of DI water to form a transparent solution, which was then transferred into a 250 ml round-bottom flask. Subsequently, 50 ml of an aqueous solution containing AlCl_3_·6H_2_O (2.41 g) was introduced dropwise into the ligand solution under mechanical stirring. The resulting mixture was subjected to reflux in an oil bath maintained at 120°C for a duration of 2 hours. After cooling down to room temperature, the white precipitate was collected and washed with DI water and methanol for several times. Finally, the MOF-303 powder was harvested after being dried under vacuum at 80°C for 12 hours. The fabrication of mMOF-303 followed a procedure similar to the synthesis of MOF-303, with the exception that 0.5 g of Fe_3_O_4_@PDA was pre-dispersed into the H_2_PZDC·H_2_O solution. The feeding masses of Fe_3_O_4_@PDA for the preparation of mMOF-303-2, mMOF-303-3 and mMOF-303-4 were 0.75 g, 1.0 g and 1.5 g, respectively.

### Materials characterization

Powder x-ray diffraction (PXRD) patterns were obtained using a Rigaku MiniFlex 600 x-ray diffraction meter (Cu Kα, 2λ = 1.5406 Å) operated at 40 kV and 15 mA. A scanning electron microscopy (SEM, JSM-7800F) and a transmission electron microscopy (TEM, JEM-2800) were used to observe the morphologies and element distribution of samples. N_2_ adsorption-desorption isotherms were measured at 77 K using a Micromeritics ASAP 2460 surface area analyzer. The thermal gravimetric (TG) analysis was conducted by a TG analyzer (Thermo plus EVO2 TG-DTA8122, Rigaku). The magnetic hysteresis curves were measured by Magnetic Property Measurement system 3 (MPMS3, Quantum Design). The thermal conductivity of samples was measured using hot-disk thermal constants analyzer (HNB-DRS2, Yutesi, China).

### Quantitative magnetic actuation characterization

Two permanent magnets with different cross-sectional areas were respectively attached to the robotic arm to drive the powder swarms across an aluminum foil substrate. The robotic arm was programmed to translate horizontally at a constant velocity of 0.1 m s^−1^ with an acceleration of 1 m s^−2^. To modulate the magnetic field intensity experienced by the mMOF-303 powder, the lift height of the robotic arm was precisely adjusted to vary the working distance between the magnet and the substrate surface. Under varied magnetic field strengths, the maximum actuated mass of the mMOF-303 powder successfully transported by each magnet was systematically recorded.

### Water adsorption/desorption measurements

The water vapor sorption isotherms were obtained by using a Belsorp Max II instrument (BEL Japan, Inc.) at 298 K. Dynamic water vapor adsorption and desorption measurements were conducted using two complementary setups depending on the sample mass. For micro-scale samples (typically a few milligrams), the measurements were carried out using a thermogravimetric (TG) analyzer coupled with a humidity generator. For larger scale samples (ranging from tens to hundreds of milligrams), the mass change during the sorption/desorption processes was monitored using a high-precision electronic balance. Prior to water adsorption measurements, all samples were heated at 120°C to remove the adsorbed water.

### Heating measurements

Photothermal measurement was conducted by a sunlight simulator (SOLAR-500, NBeT, China) and an optical filter for the standard AM 1.5 G spectrum was used as a light source. The intensity of solar irradiation was regulated to 1 kW m^−2^ by a solar power meter (TM-207, TENMARS, China). For electrothermal tests, the samples were heated by a heating plate, with the temperature controlled by the voltage. The magnetothermal heating process was driven by a compact electromagnetic heating device (fig. S32). The heating temperature was monitored with an infrared imager (Testo 883, Germany).

### Humidity regulation measurements

During the humidity regulation process, the relative humidity was monitored using a humidity sensor with an accuracy of ±2% RH and a resolution of 0.1% RH. The electrical heating plate used was rated at 12 V and 5 W, with the actual operating temperature controlled by the applied voltage (power). The mass of mMOF-303 used for humidity regulation was 100 mg, which was preheated at 80°C for 2 hours prior to use.
