# Supplementary material for: Swarm-inspired magnetic metal-organic framework-based microbots for on-demand moisture manipulation
Source: Sci Adv. 2026 Nov 13;12(46):eaeg6193. doi: 10.1126/sciadv.aeg6193 (PMC13763934; doi:10.1126/sciadv.aeg6193)
Supplement: Supplementary file 1 [file sciadv.aeg6193_sm.pdf]

Science Advances
AAAS

Supplementary Materials for

Swarm-inspired magnetic metal-organic framework-based microbots for on-demand moisture manipulation

Mingren Cheng *et al.*

Corresponding author: Moyuan Cao, mycao@nankai.edu.cn; Xian-He Bu, buxh@nankai.edu.cn

Sci. Adv. **12**, eaeg6193 (2026)
DOI: 10.1126/sciadv.aeg6193

The PDF file includes:

Figs. S1 to S39
Tables S1 and S2
Legends for movies S1 to S9

Other Supplementary Material for this manuscript includes the following:

Movies S1 to S9

Supplementary Text

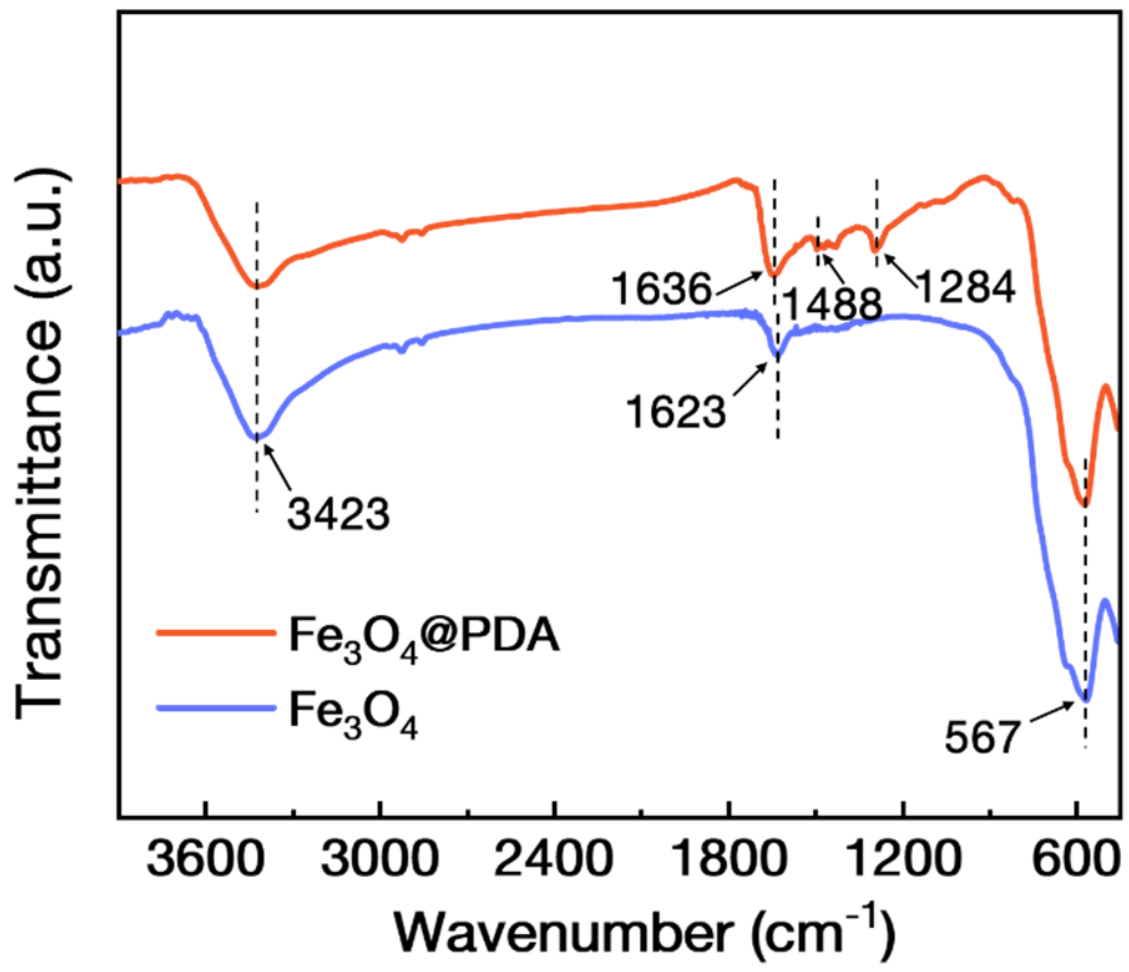

Fig. S1.

FTIR spectra of Fe_3O_4 and Fe_3O_4@PDA.

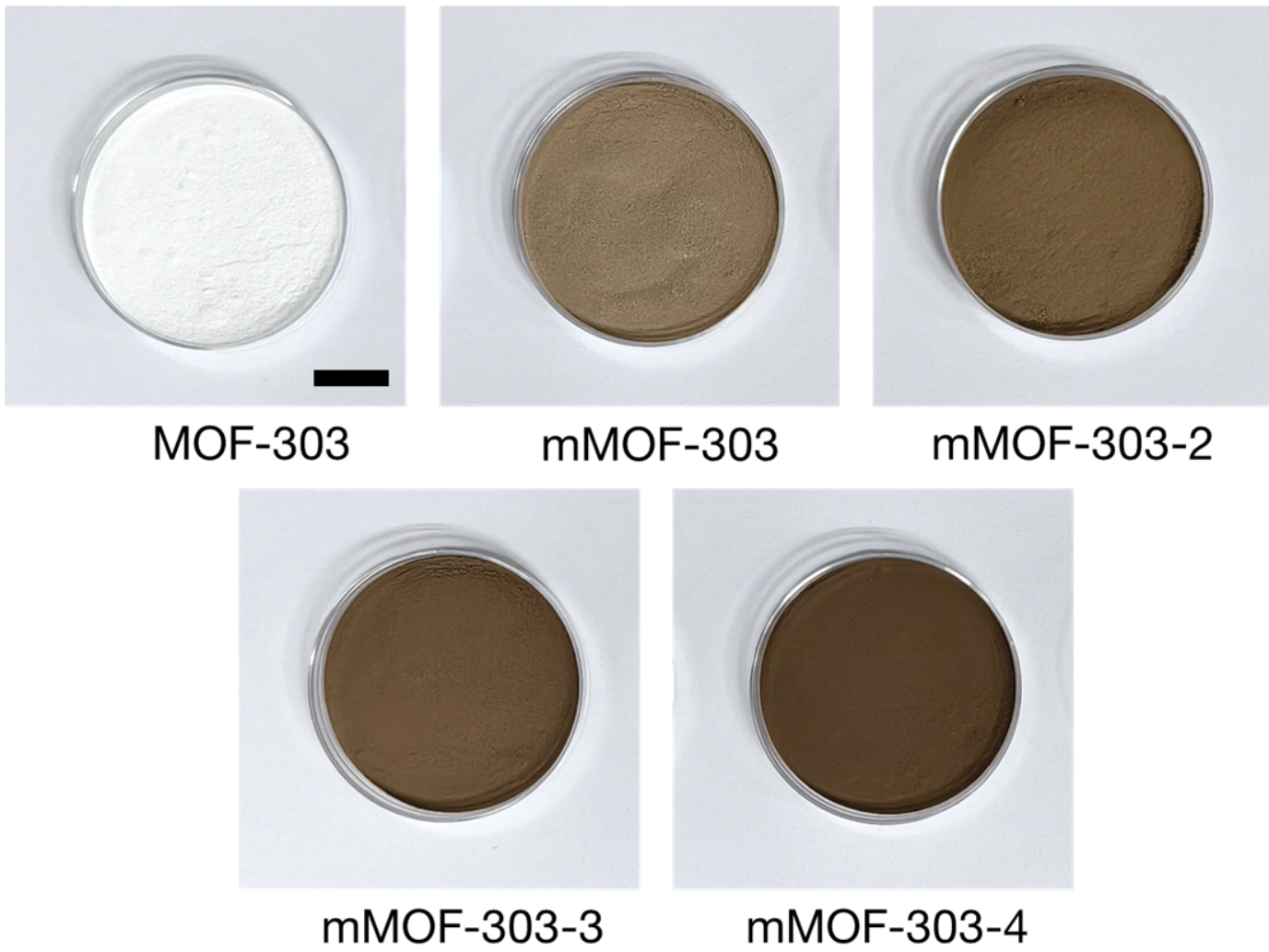

Fig. S2.

Photographs of MOF-303, mMOF-303, mMOF-303-2, mMOF-303-3 and mMOF-303-4 powders. Scale bar, 1 cm.

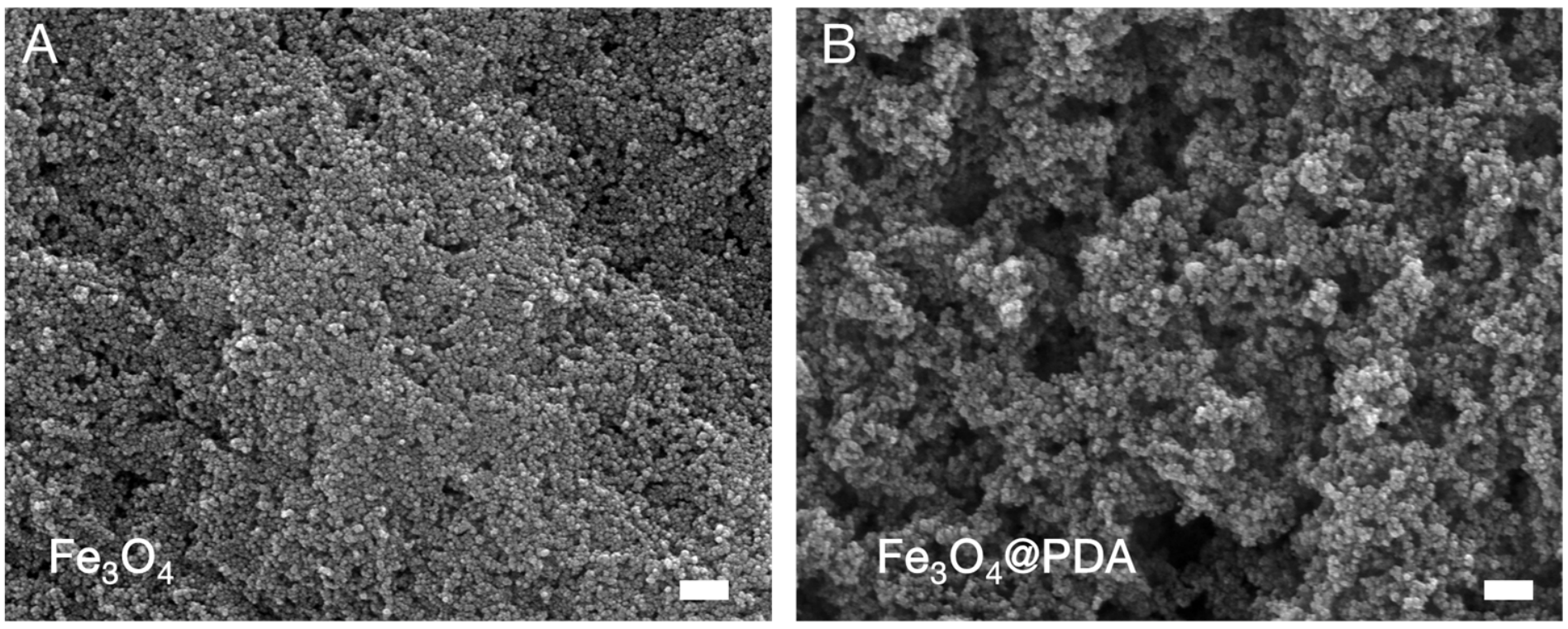

Fig. S3.
SEM images of (A) Fe_3O_4 and (B) Fe_3O_4@PDA. Scale bars, 200 nm.

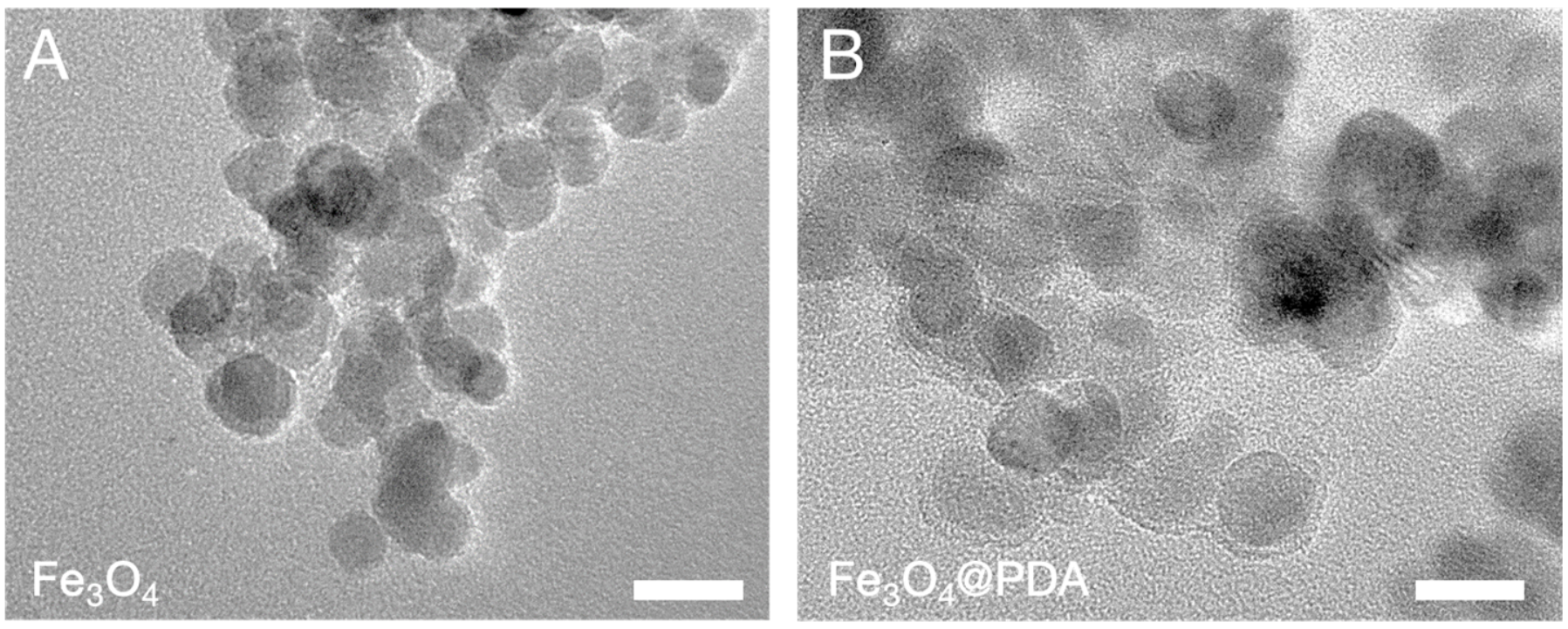

Fig. S4.
TEM images of (A) Fe_3O_4 and (B) Fe_3O_4@PDA. Scale bars, 20 nm.

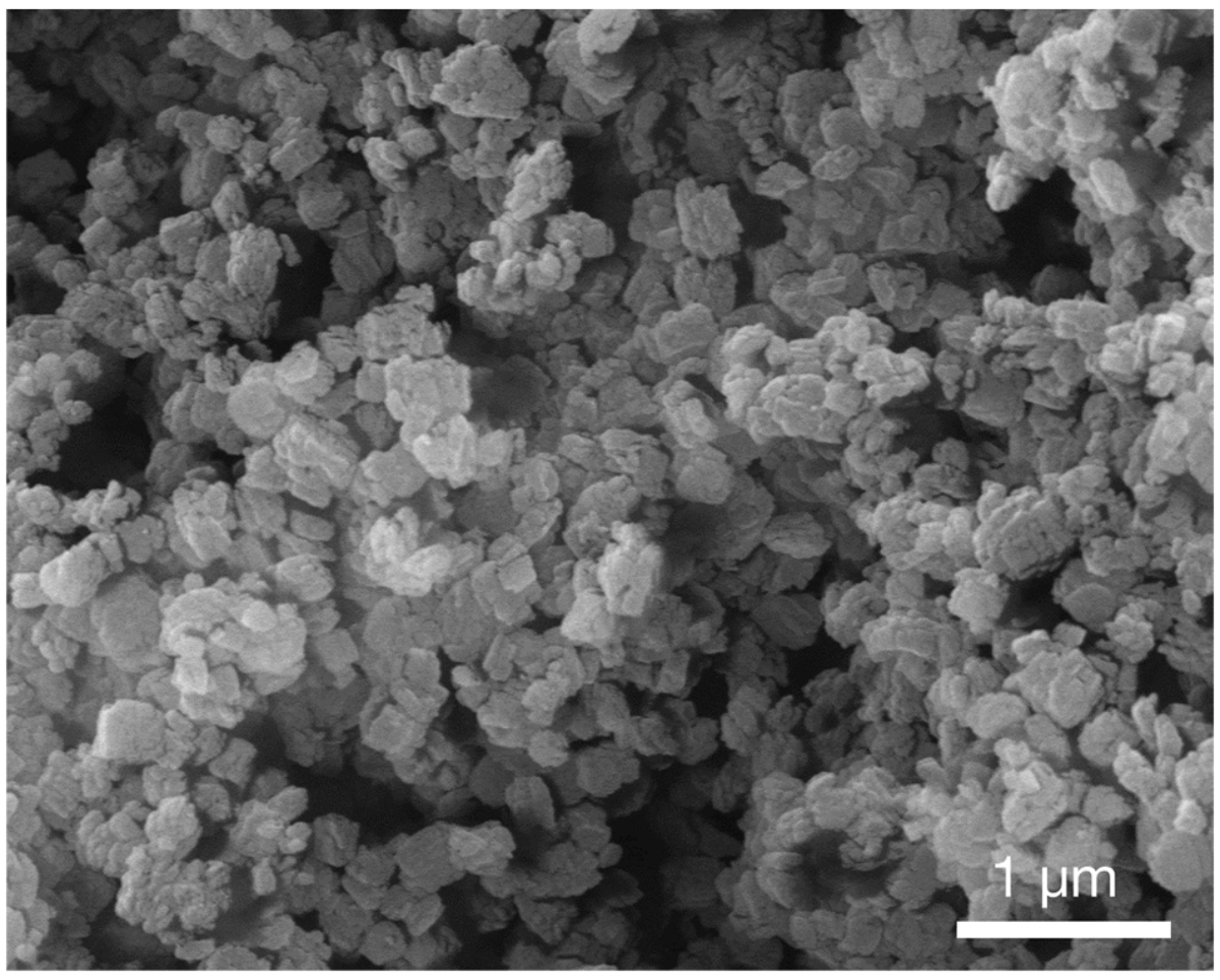

Fig. S5.
SEM image of pure MOF-303.

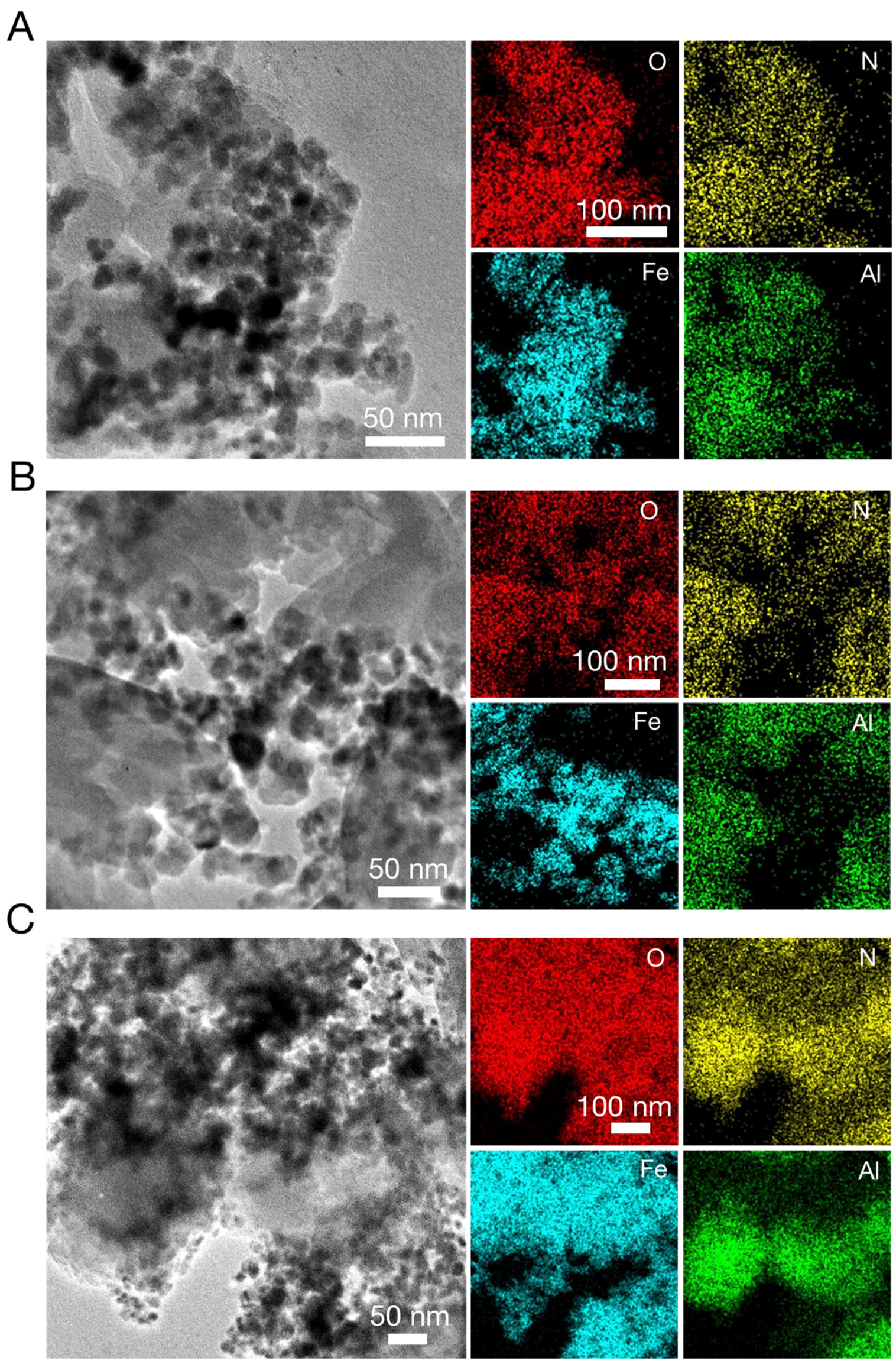

Fig. S6.

TEM images and corresponding elemental mapping images of (A) mMOF-303-2, (B) mMOF-303-3 and (C) mMOF-303-4.

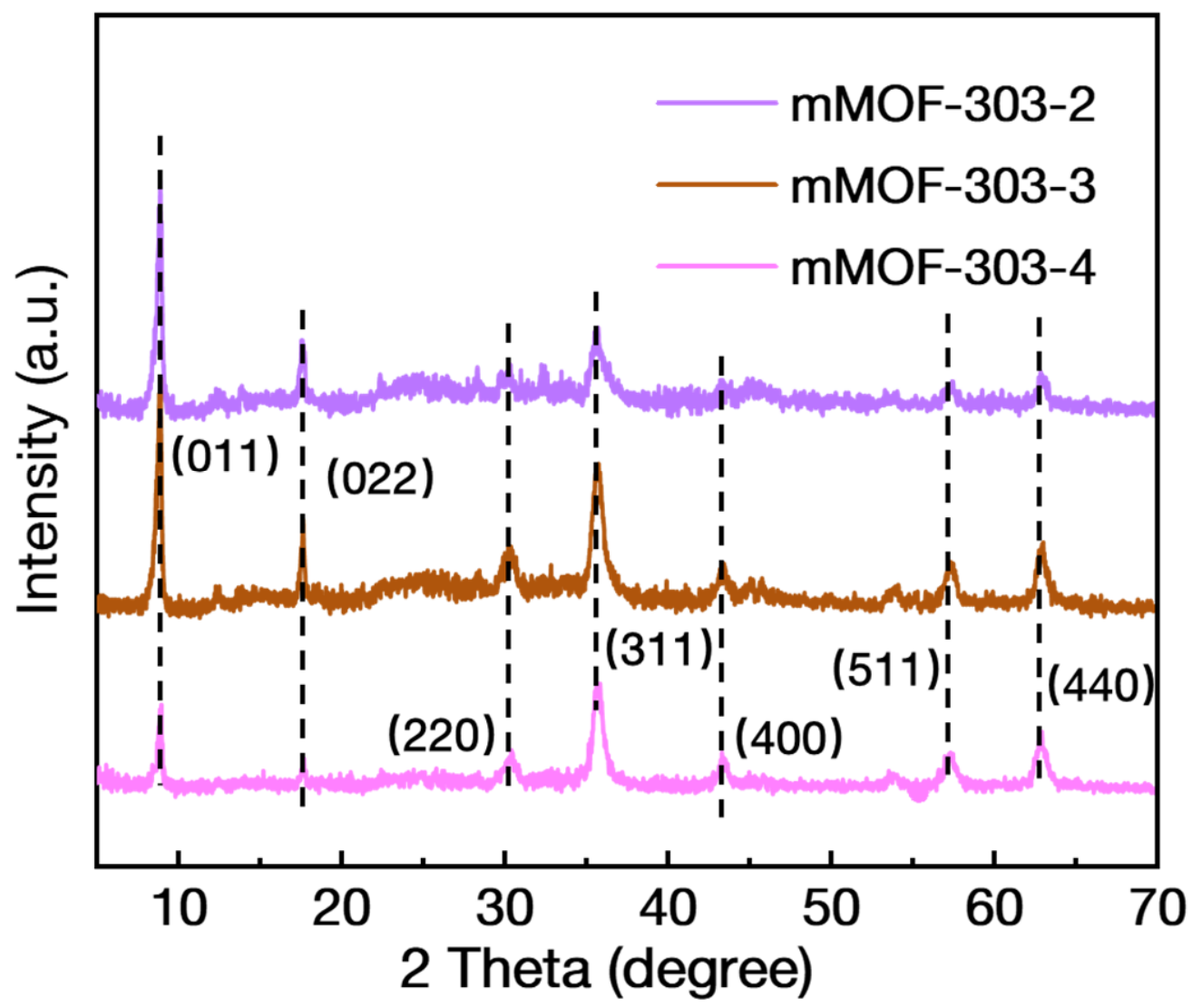

Fig. S7.
PXRD patterns of mMOF-303-2, mMOF-303-3 and mMOF-303-4.

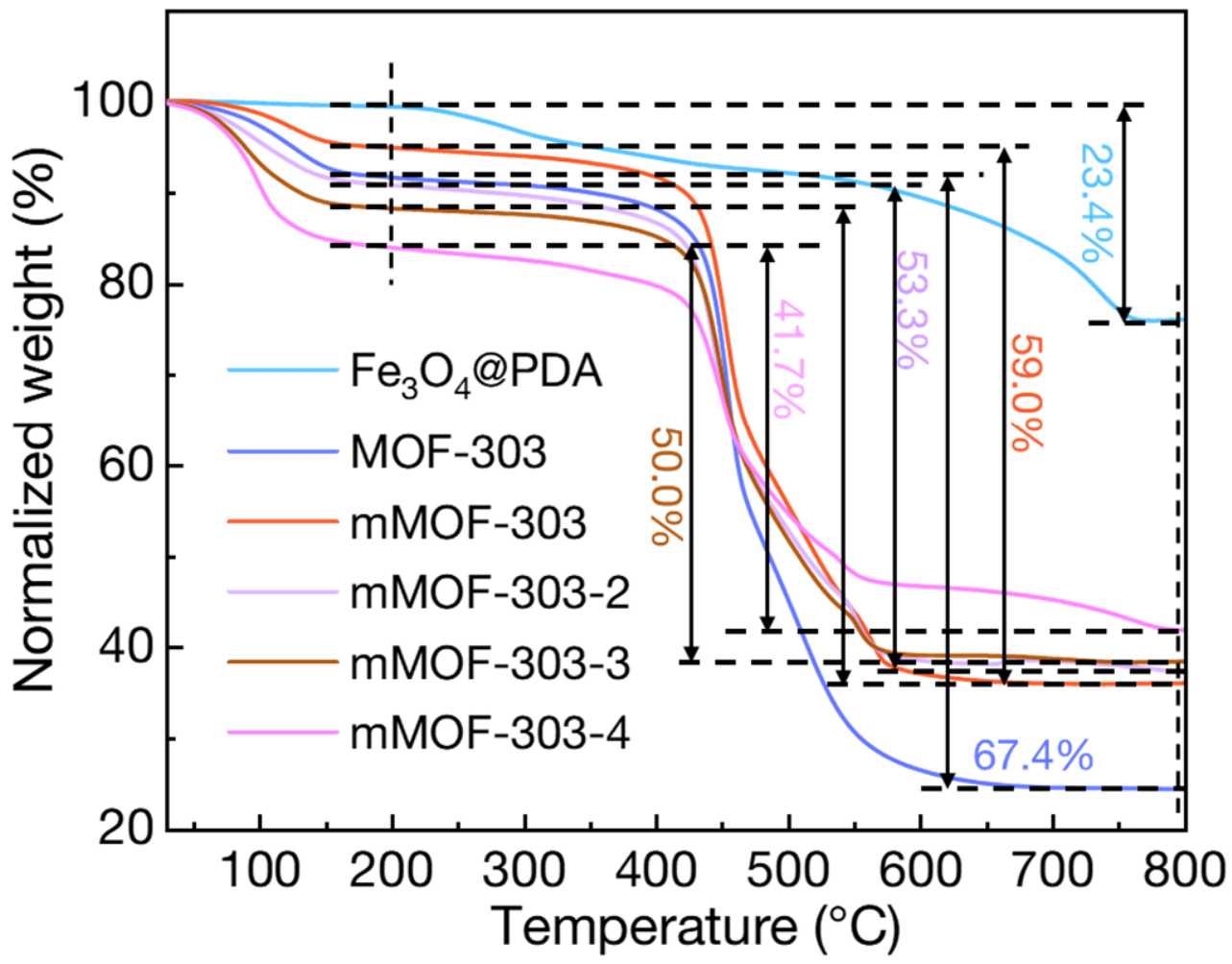

Fig. S8.

TG curves of MOF-303, Fe_3O_4@PDA, mMOF-303, mMOF-303-2, mMOF-303-3 and mMOF-303-4.

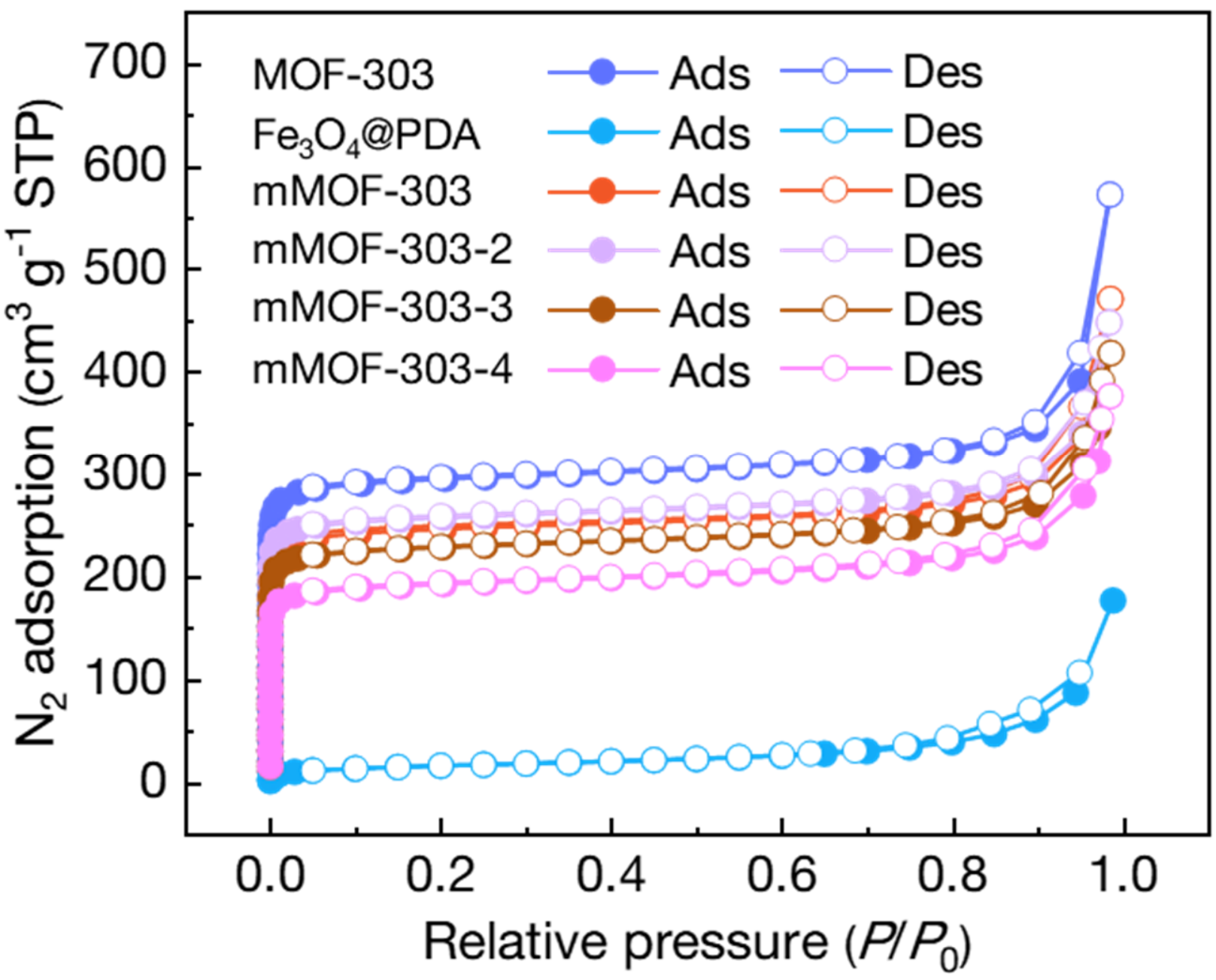

Fig. S9.

N_2 sorption isotherms at 77 K of Fe_3O_4@PDA, MOF-303, mMOF-303, mMOF-303-2, mMOF-303-3 and mMOF-303-4. The corresponding BET specific surface areas are 57.0, 1666.9, 1178.8, 1028.0, 909.5 and 762.0 $m^2 g^{-1}$, respectively.

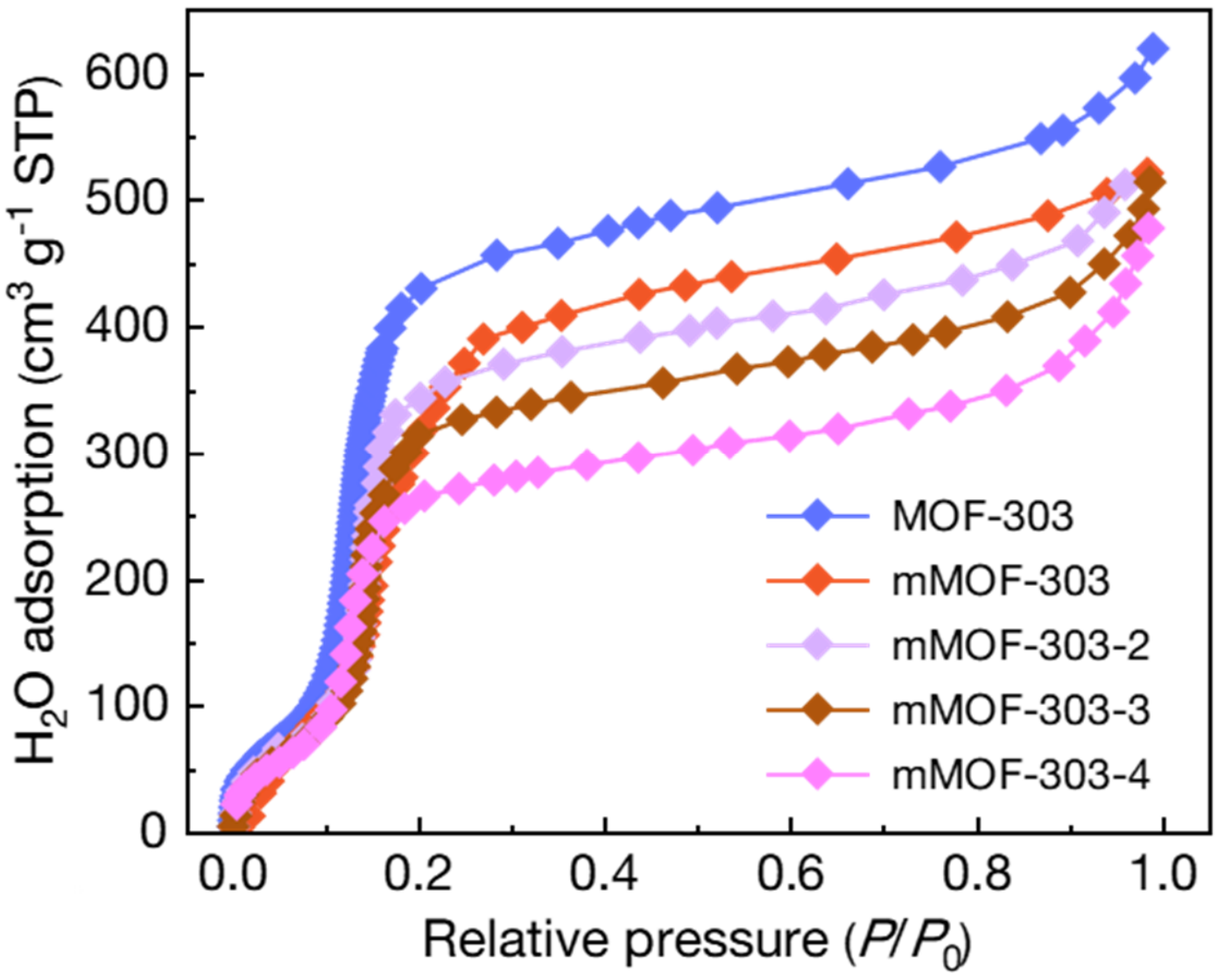

Fig. S10.

Water sorption isotherms of MOF-303, mMOF-303, mMOF-303-2, mMOF-303-3 and mMOF-303-4.

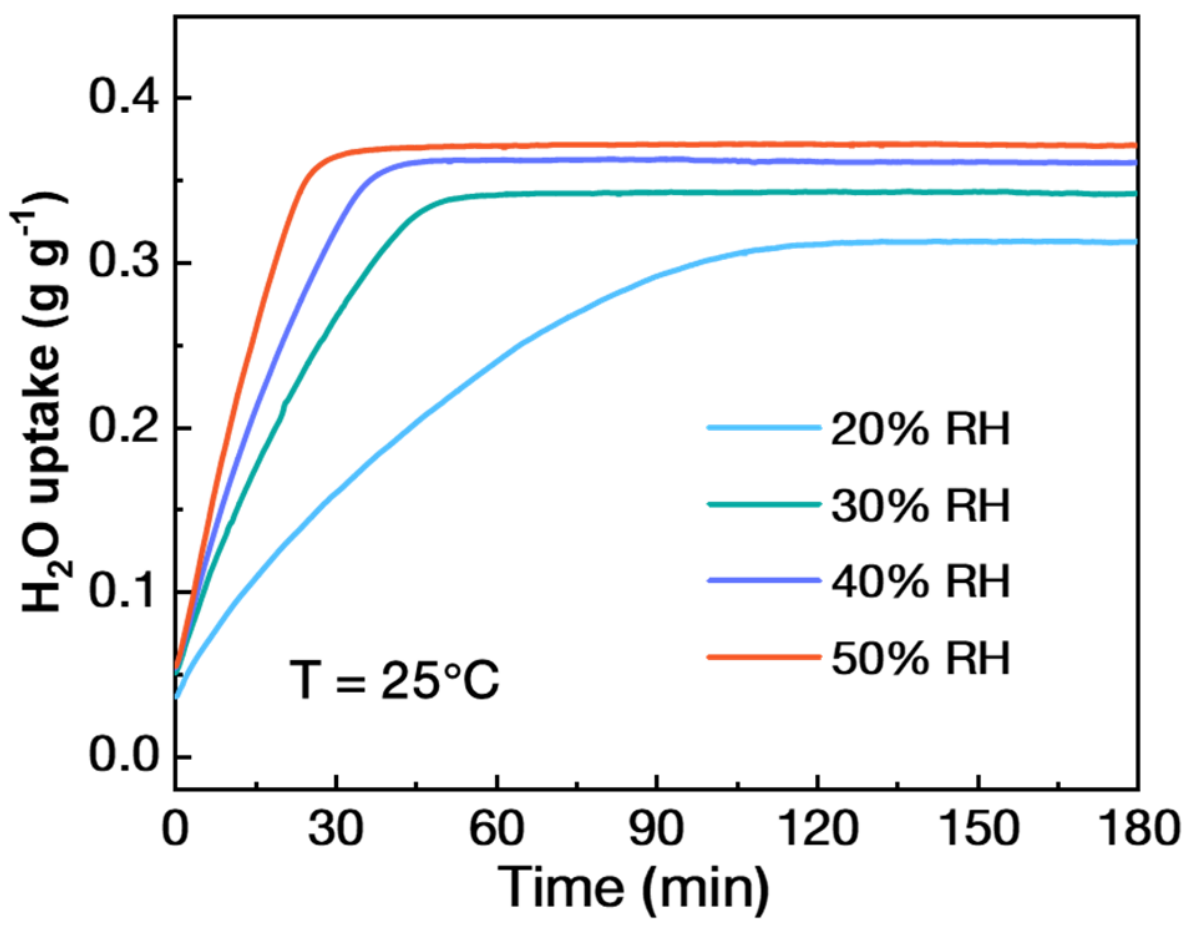

Fig. S11.

Dynamic water uptake curves of mMOF-303 at different RHs using a TG analyzer combined with a humidity generator.

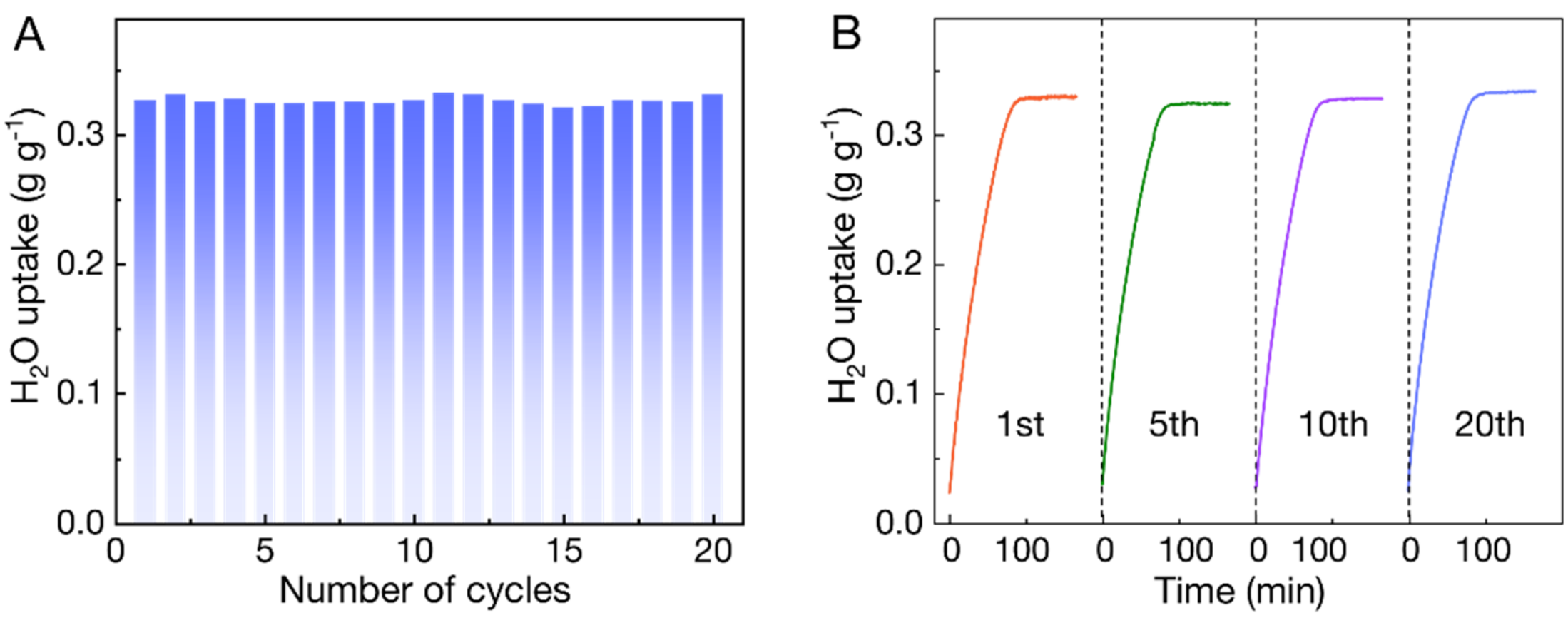

Fig. S12.

Adsorption-desorption cycling tests of mMOF-303 at 25% RH. (A) Adsorption capacities and **(B)** dynamic water uptake curves during adsorption-desorption cycles.

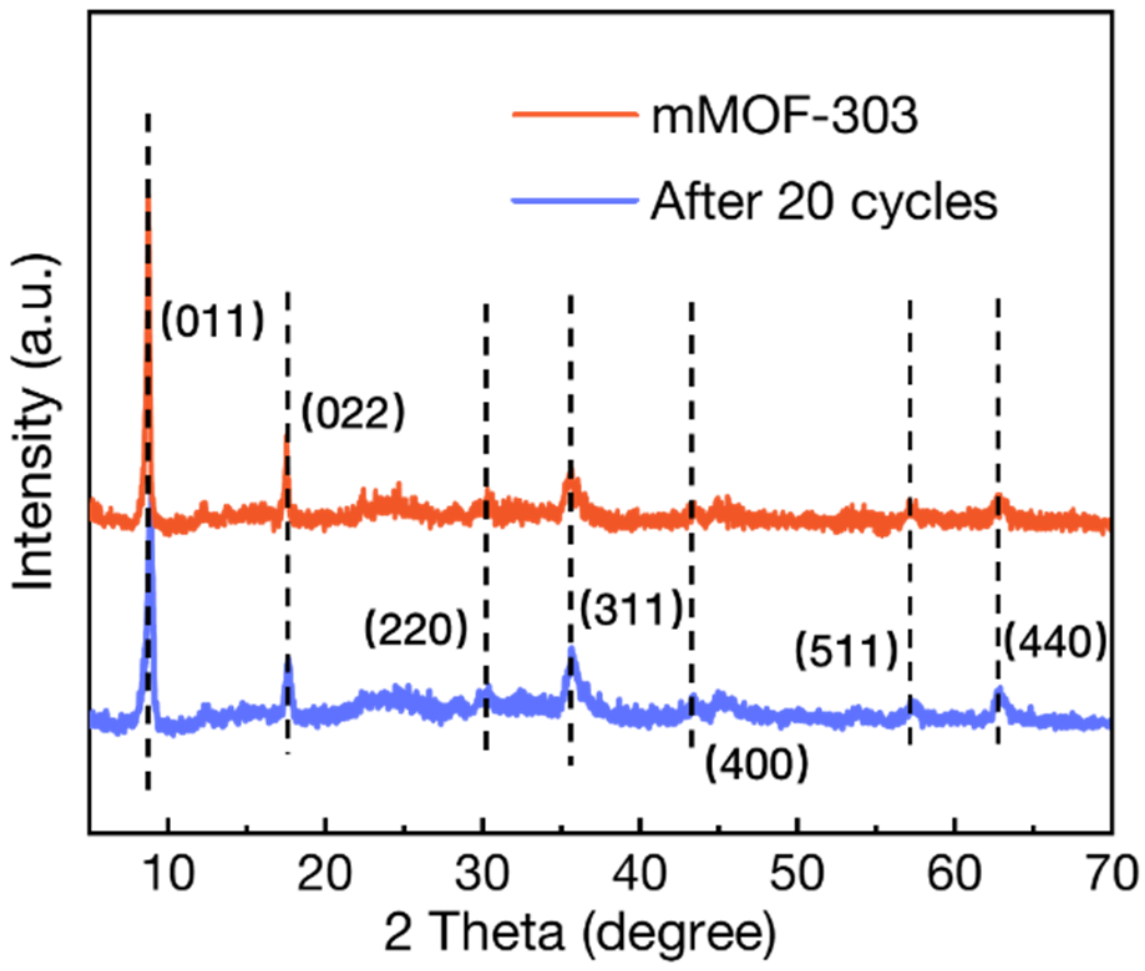

Fig. S13.
PXRD patterns of mMOF-303 before and after 20 cycles.

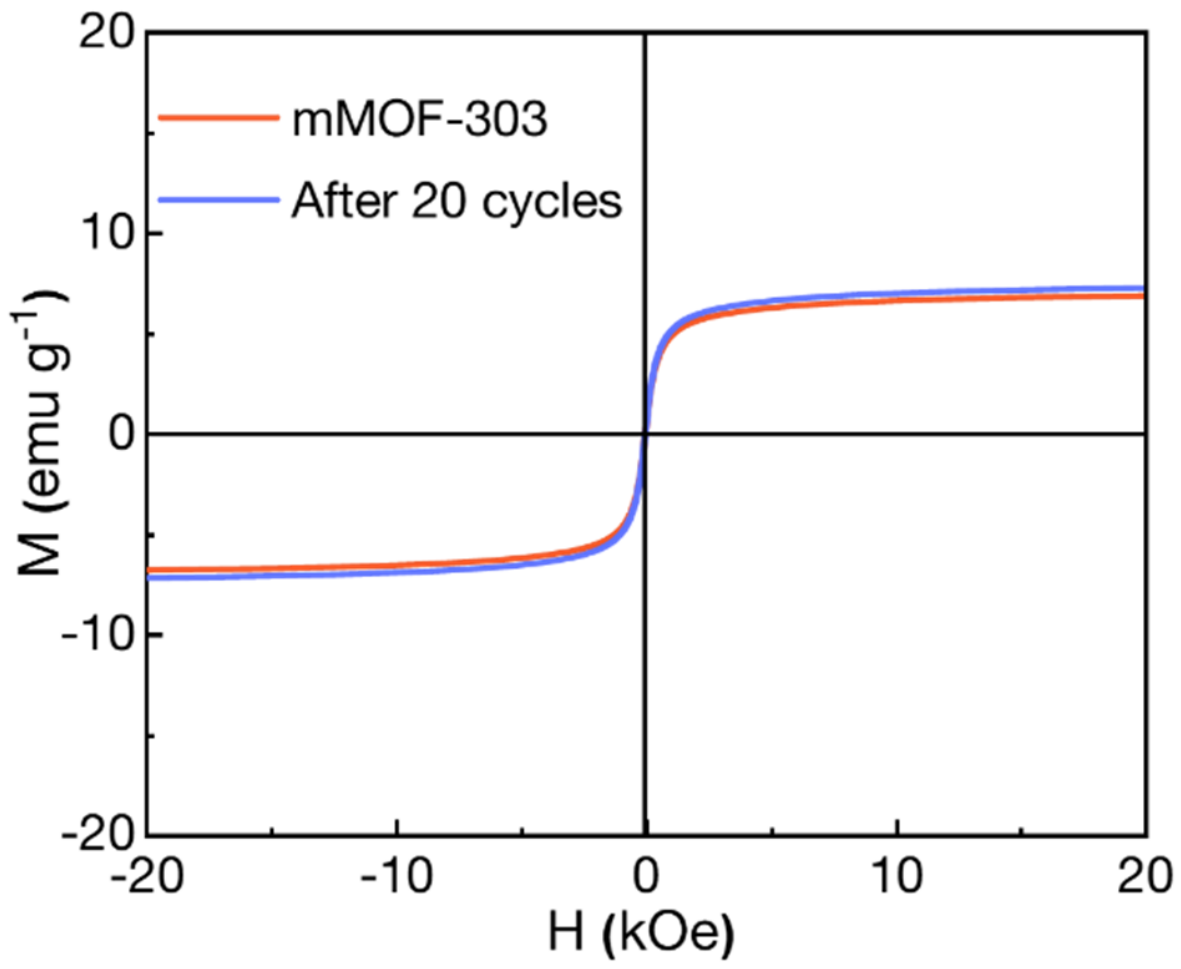

Fig. S14.
Magnetic hysteresis loop of mMOF-303 before and after 20 cycles.

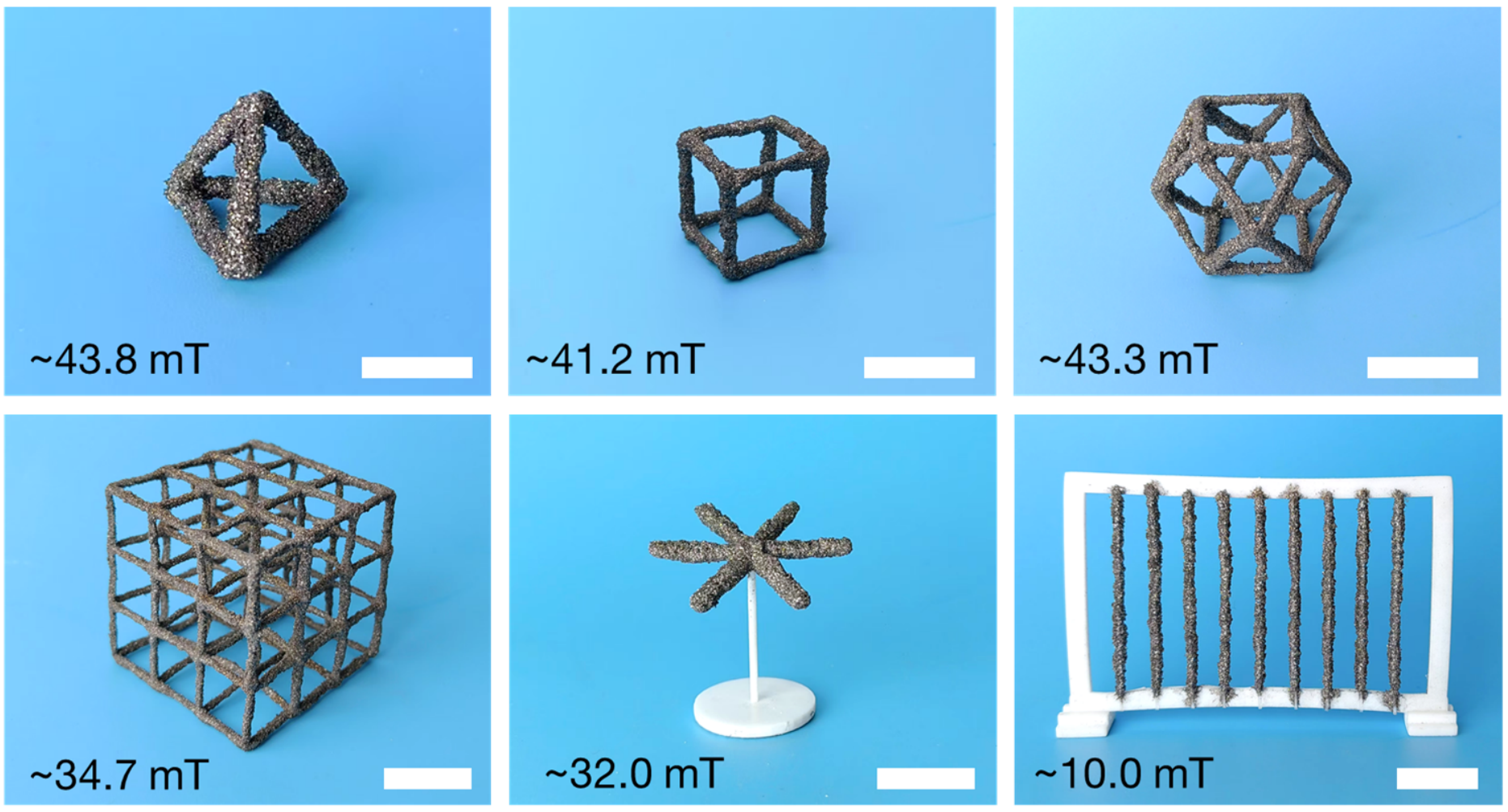

Fig. S15.
Photographs of various shaped magnetic substrates. Scale bars, 1 cm.

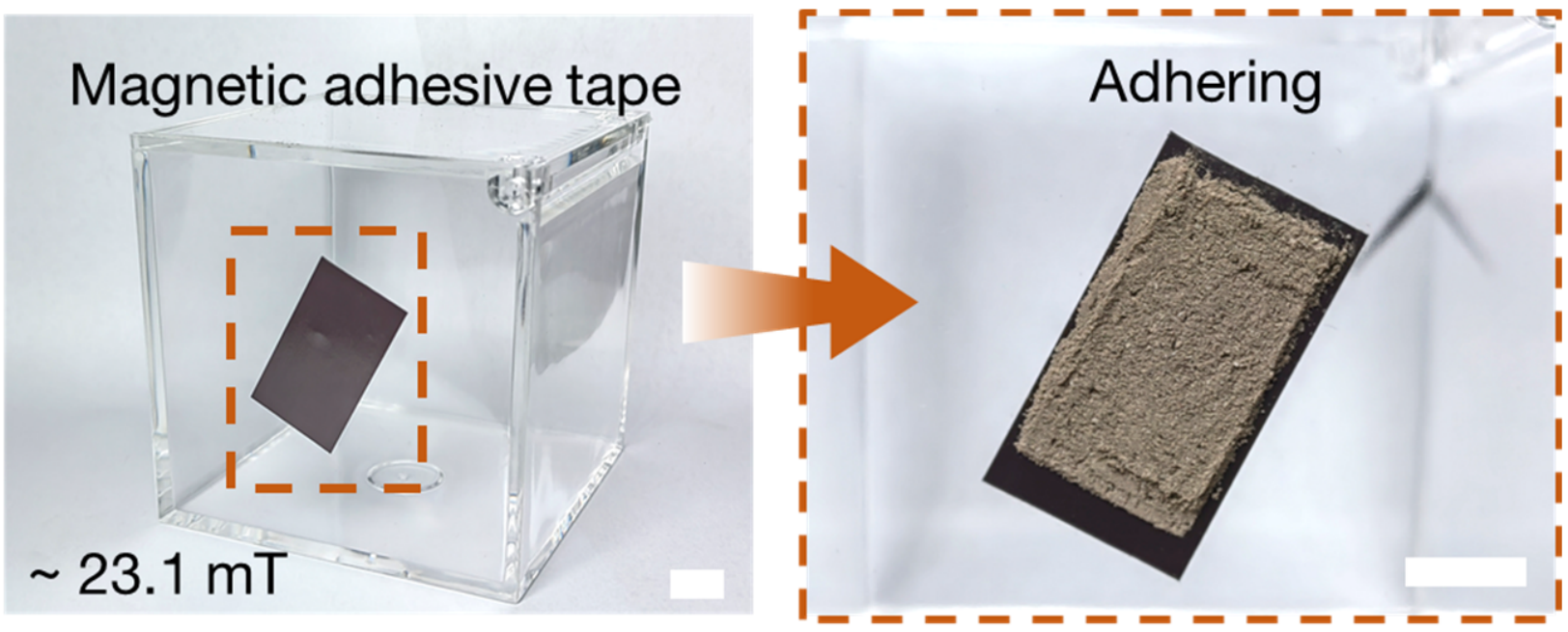

Fig. S16.
Adhesion of mMOF-303 to object surface via magnetic adhesive tape. Scale bars, 1 cm.

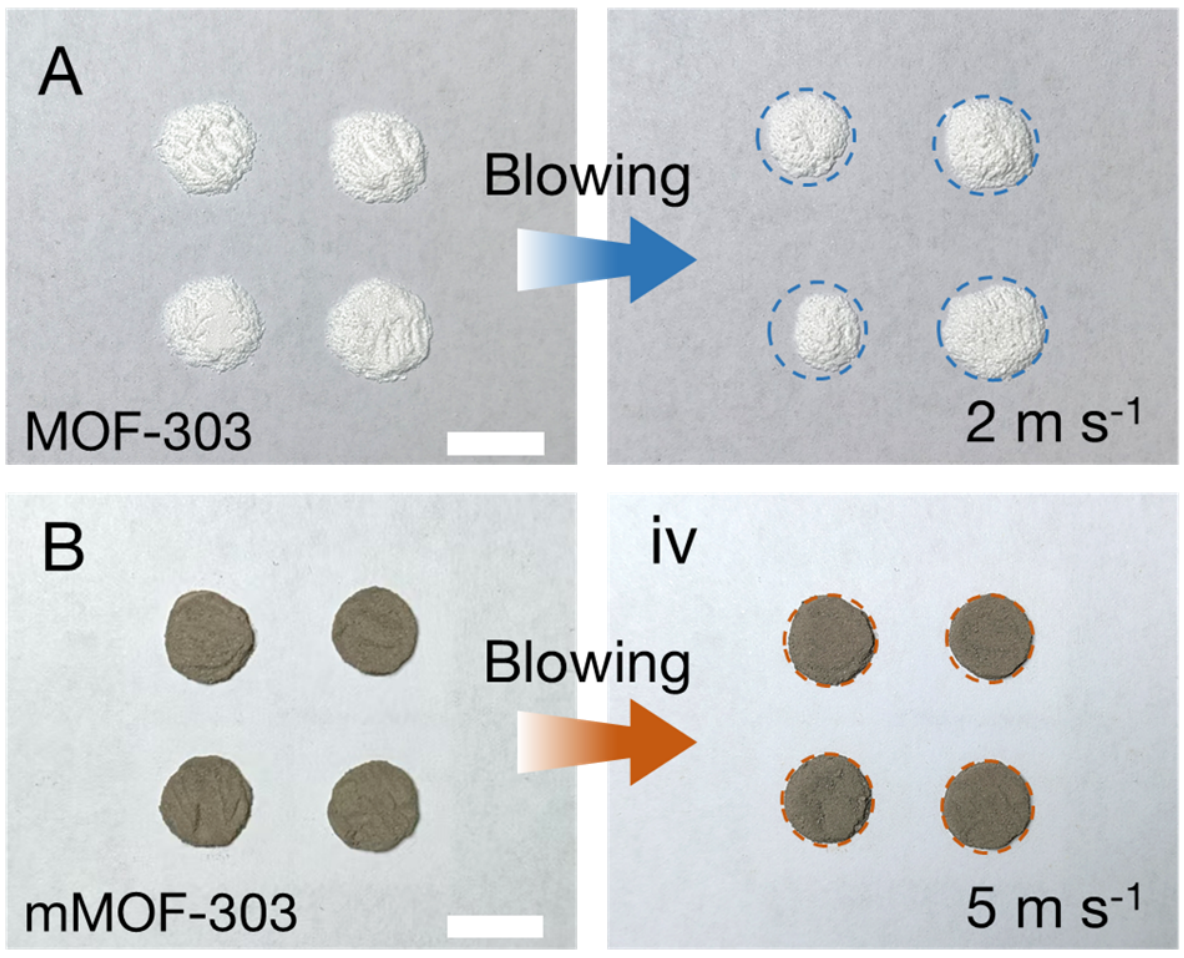

Fig. S17.

Blowing test for evaluating purging resistance. Comparison of purging resistance between **(A)** MOF-303 and **(B)** magnetically adhered mMOF-303 powder. Scale bars, 1 cm. The applied magnetic field strength is ~ 502.7 mT.

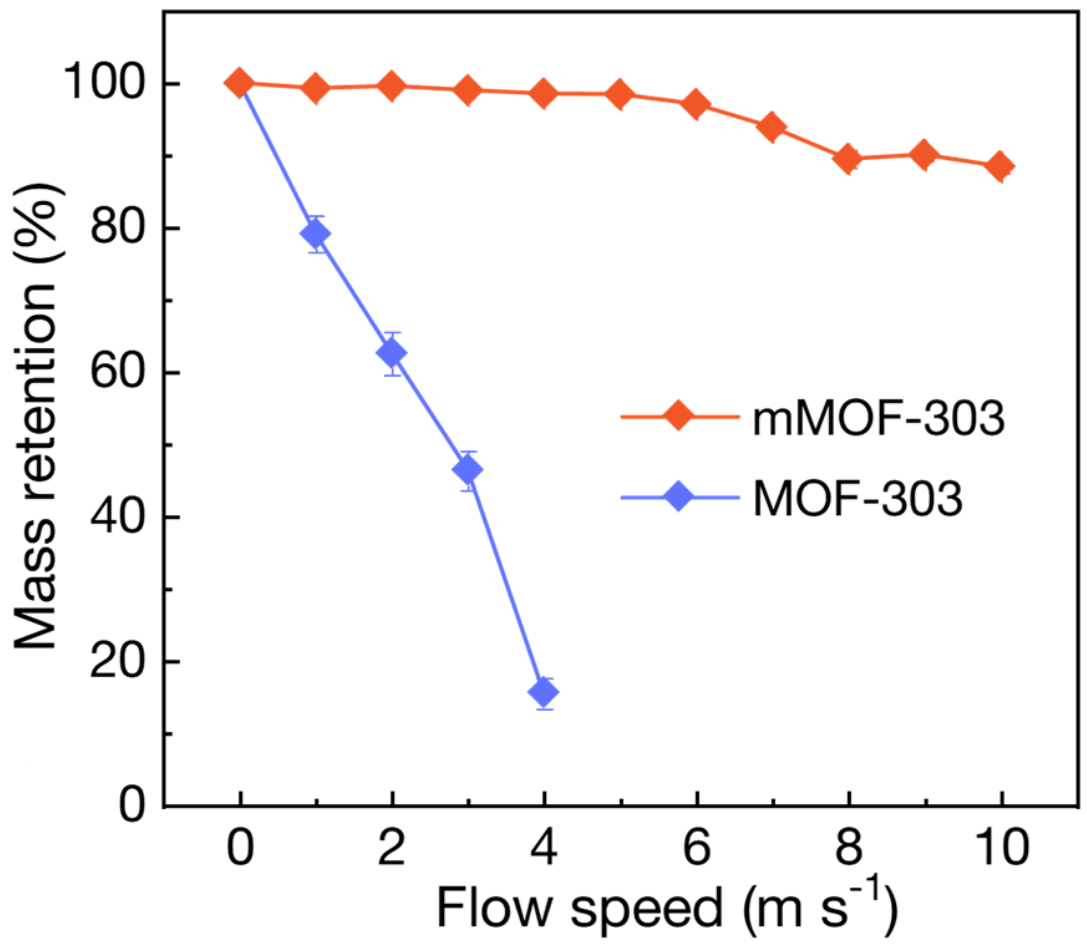

Fig. S18.
Quantitative blowing test for MOF-303 and mMOF-303 powders.

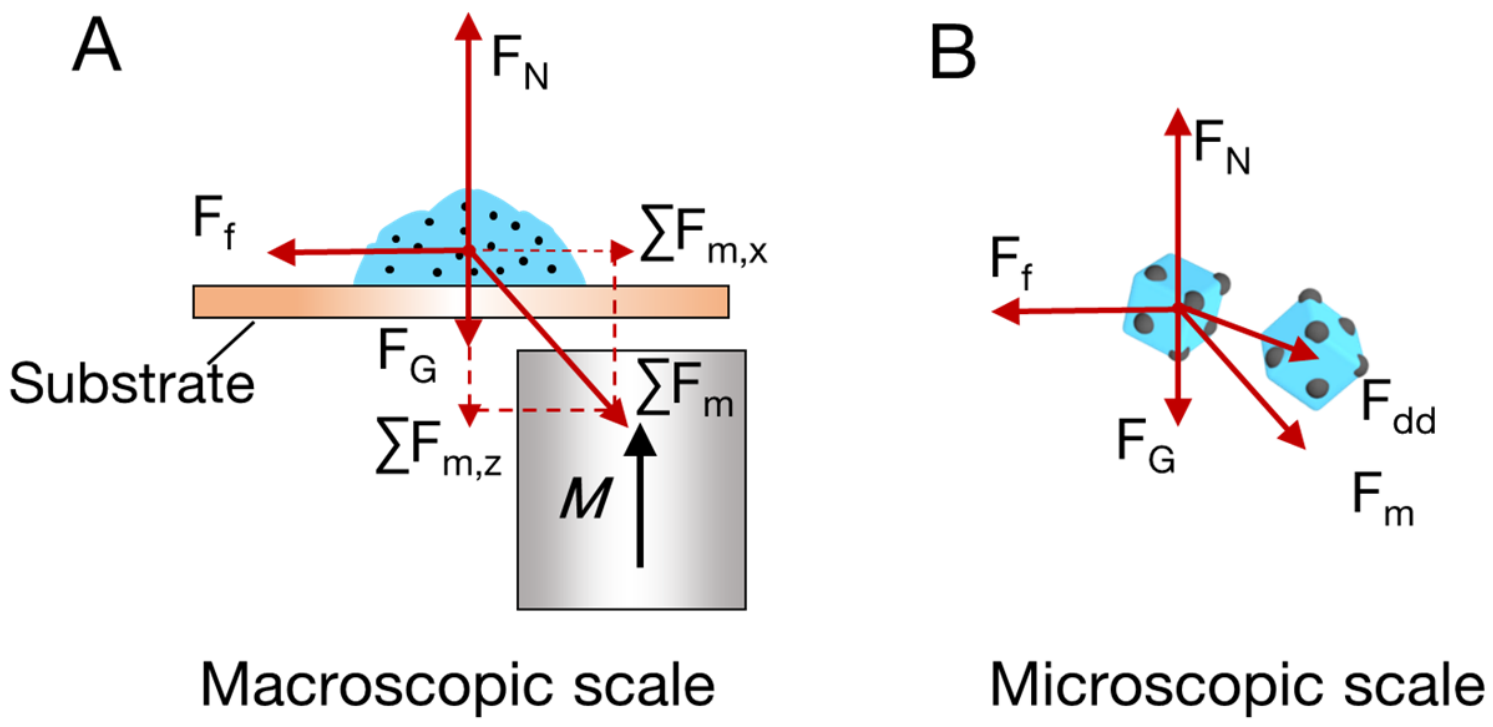

Fig. S19.

Schematic force analysis of mMOF-303 during motion. (A) Macroscopic force behavior of the powder collective and **(B)** microscopic particle interactions.

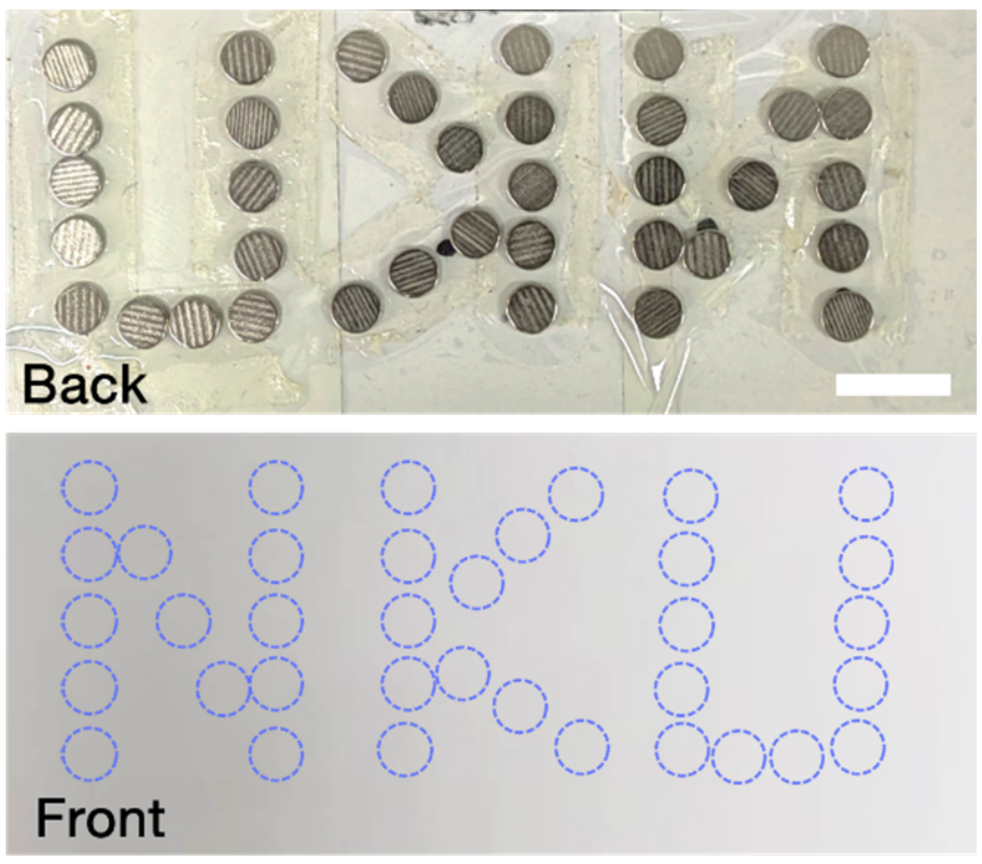

Fig. S20.

Front and back views of the programmable magnetic substrate patterned with specific letters. Scale bar, 2 cm. The applied magnetic field strength is ~ 53.3 mT.

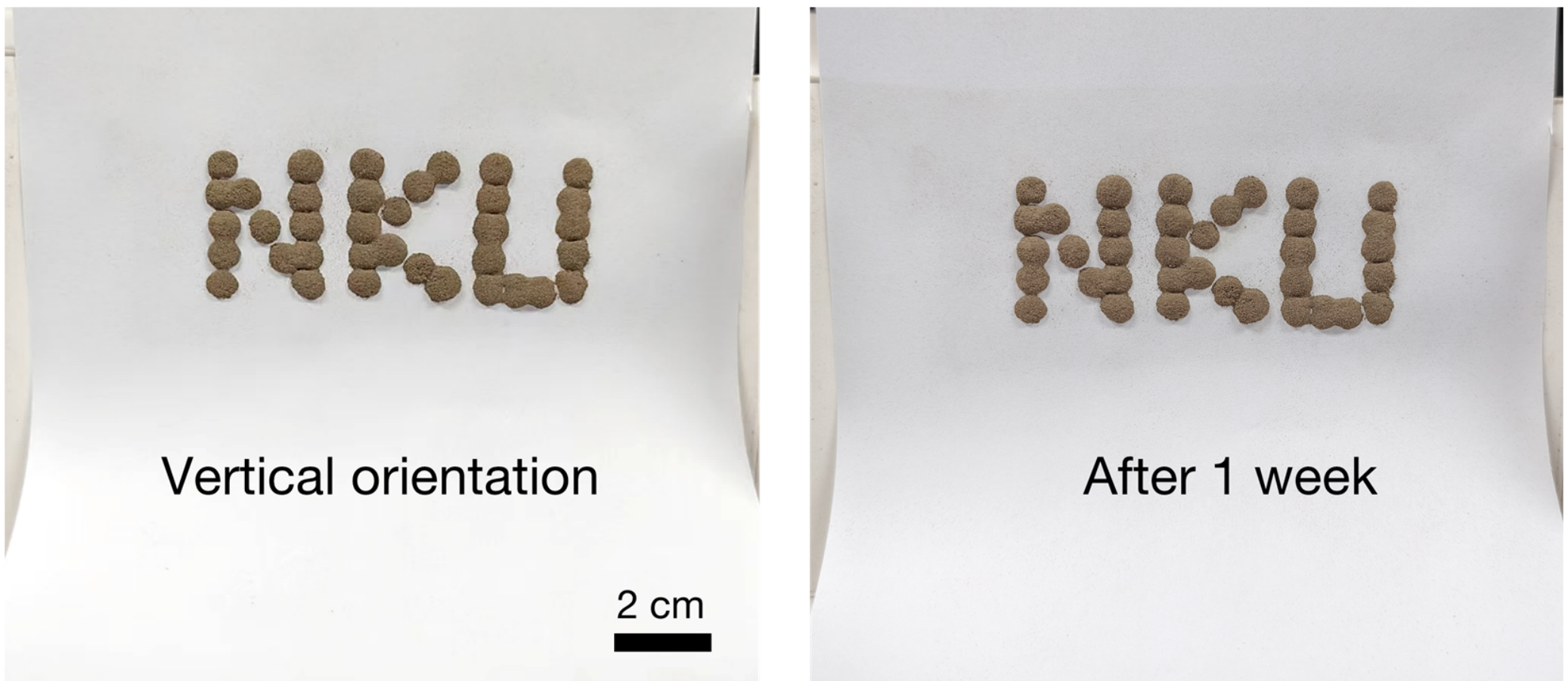

Fig. S21.

Long-term structural stability of vertically oriented mMOF-303 magnetically molded patterns over 1 week.

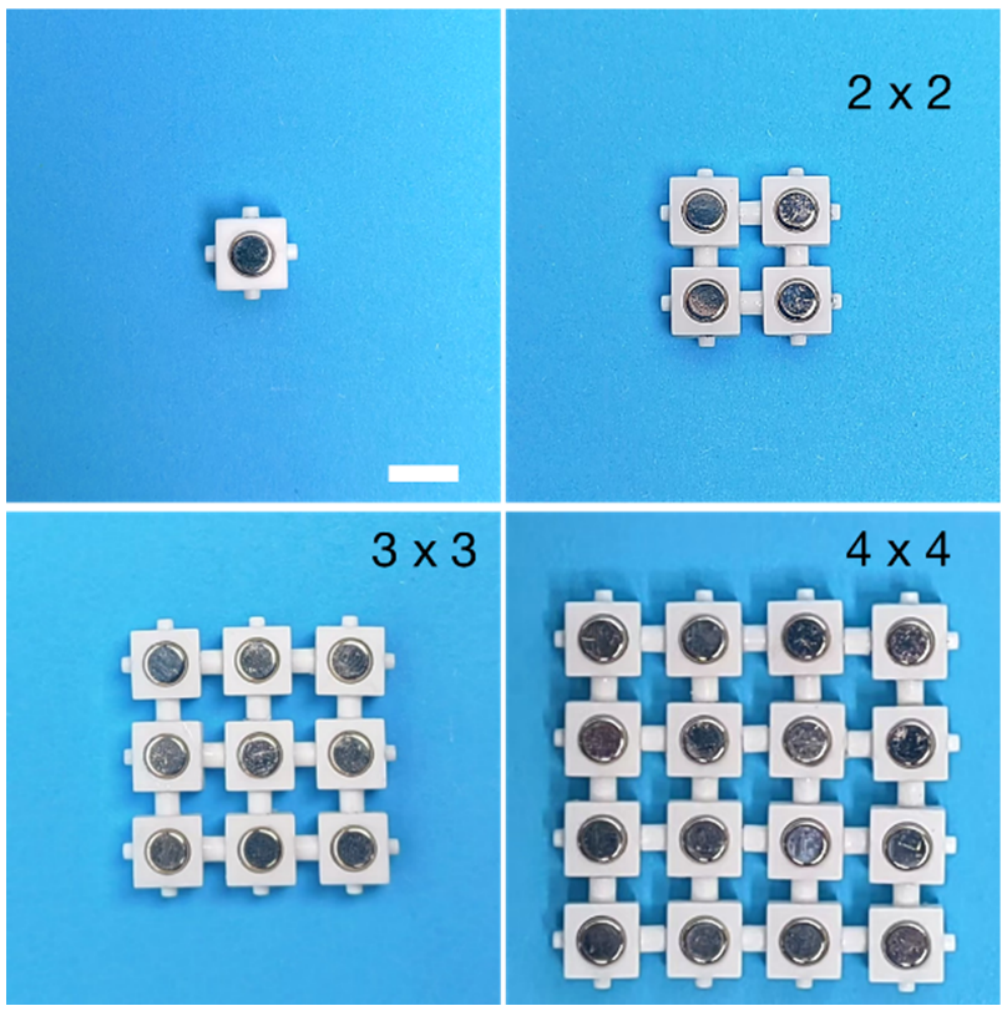

Fig. S22.

Modular assembly of individual magnetic units. Scale bars, 1 cm. The applied magnetic field strength is ~ 525.5 mT.

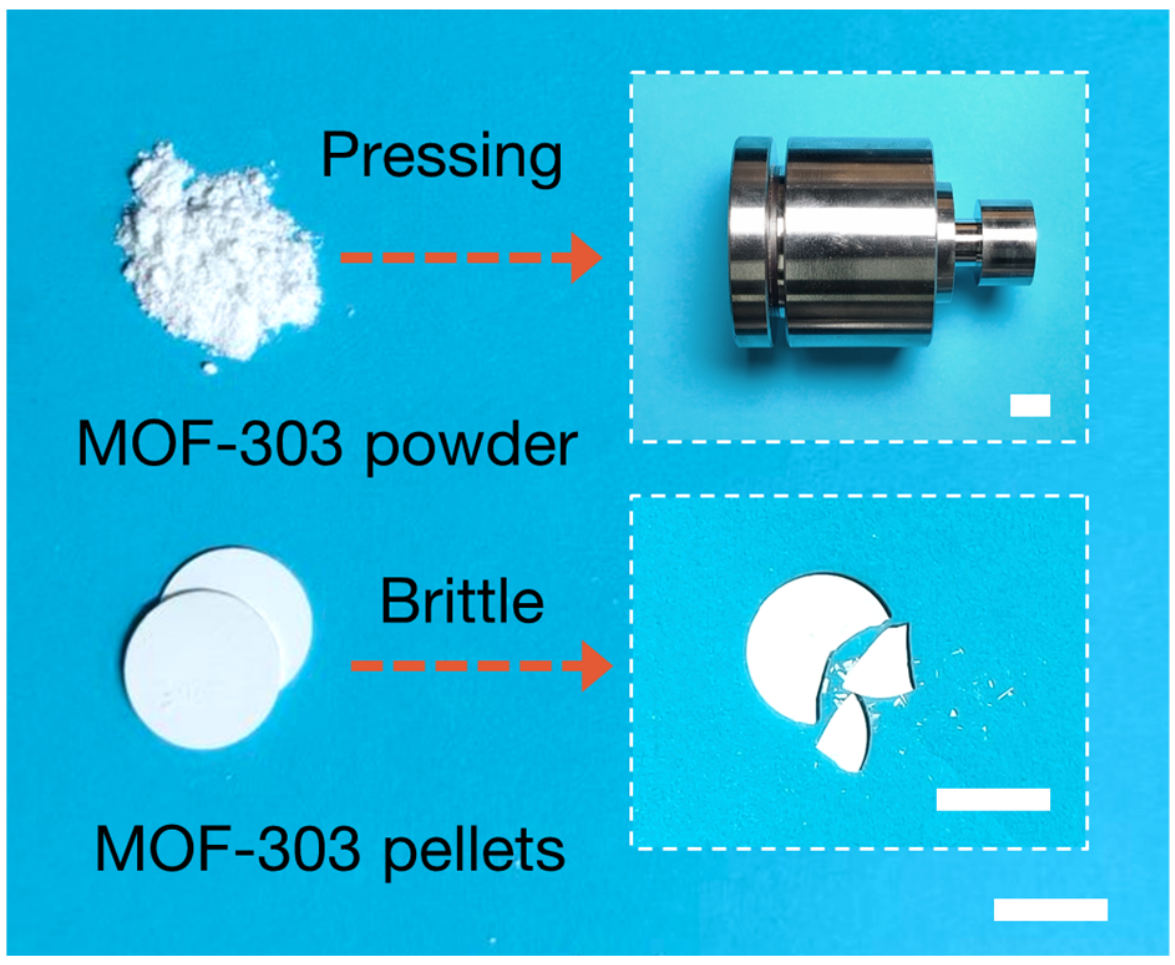

Fig. S23.

Photographs of MOF-303 pellets formed by compression molding and their brittle nature. Scale bars, 1 cm.

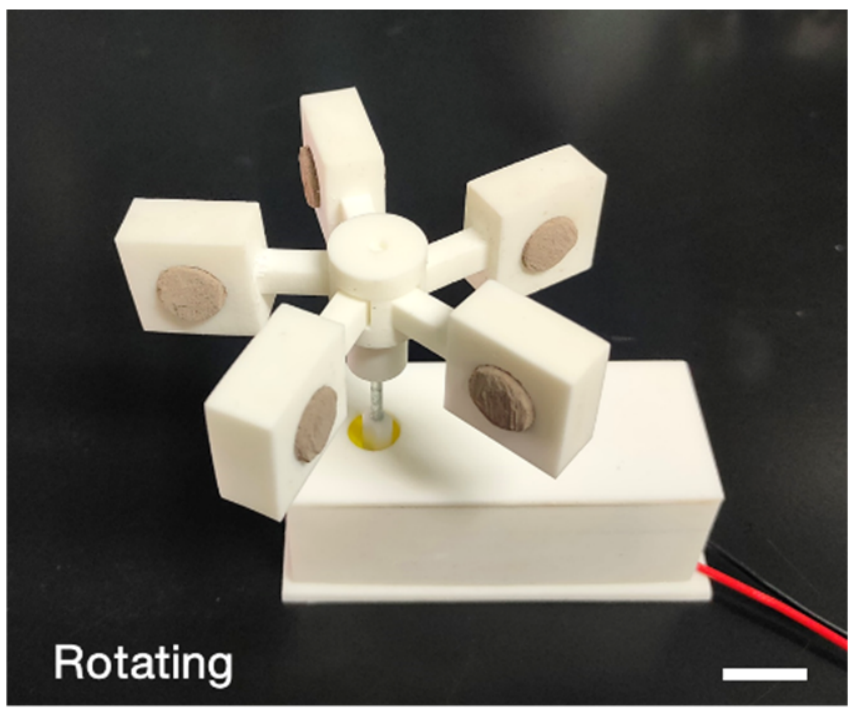

Fig. S24.

Photograph of the testing setup for water adsorption of mMOF-303 rotating with it. Sample loading is 50 mg cm^{-2}. Scale bars, 1 cm. The applied magnetic field strength is ~ 525.5 mT.

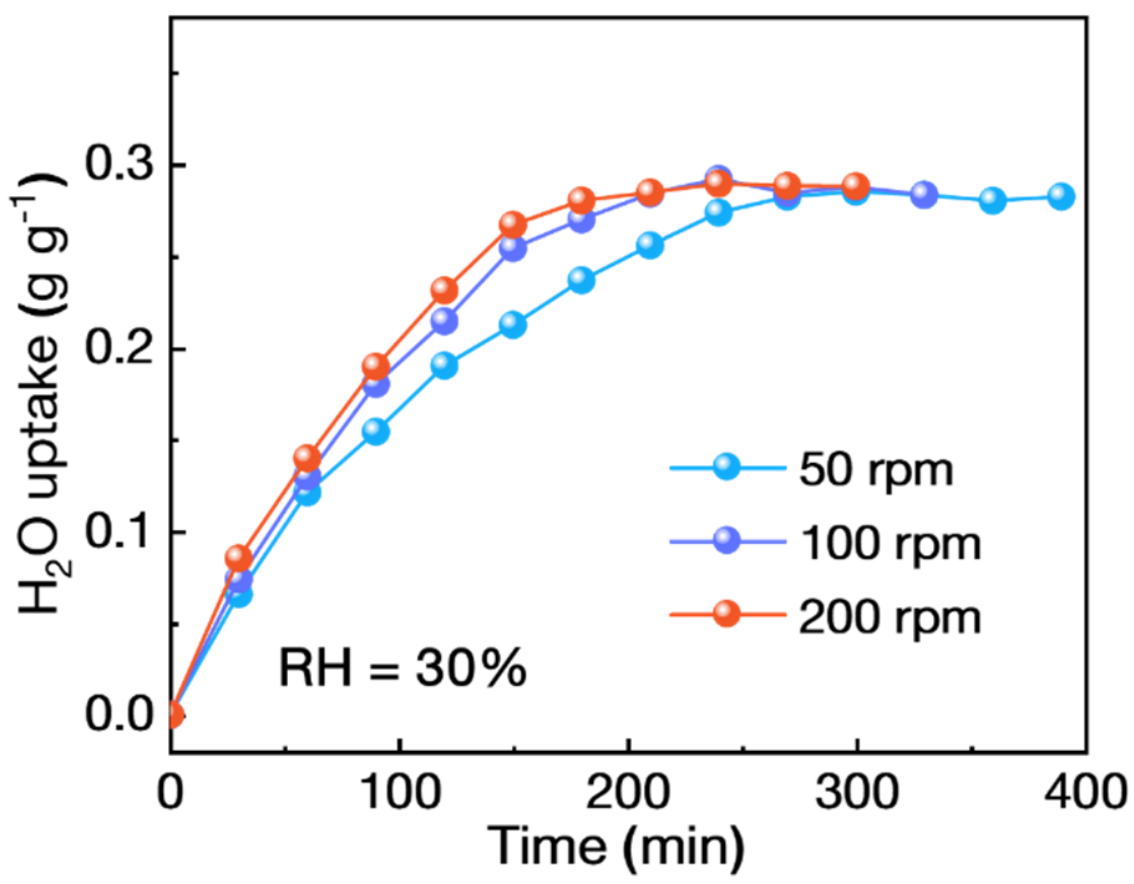

Fig. S25.
Dynamic water uptake curves of mMOF-303 at different rotation speeds (30% RH).

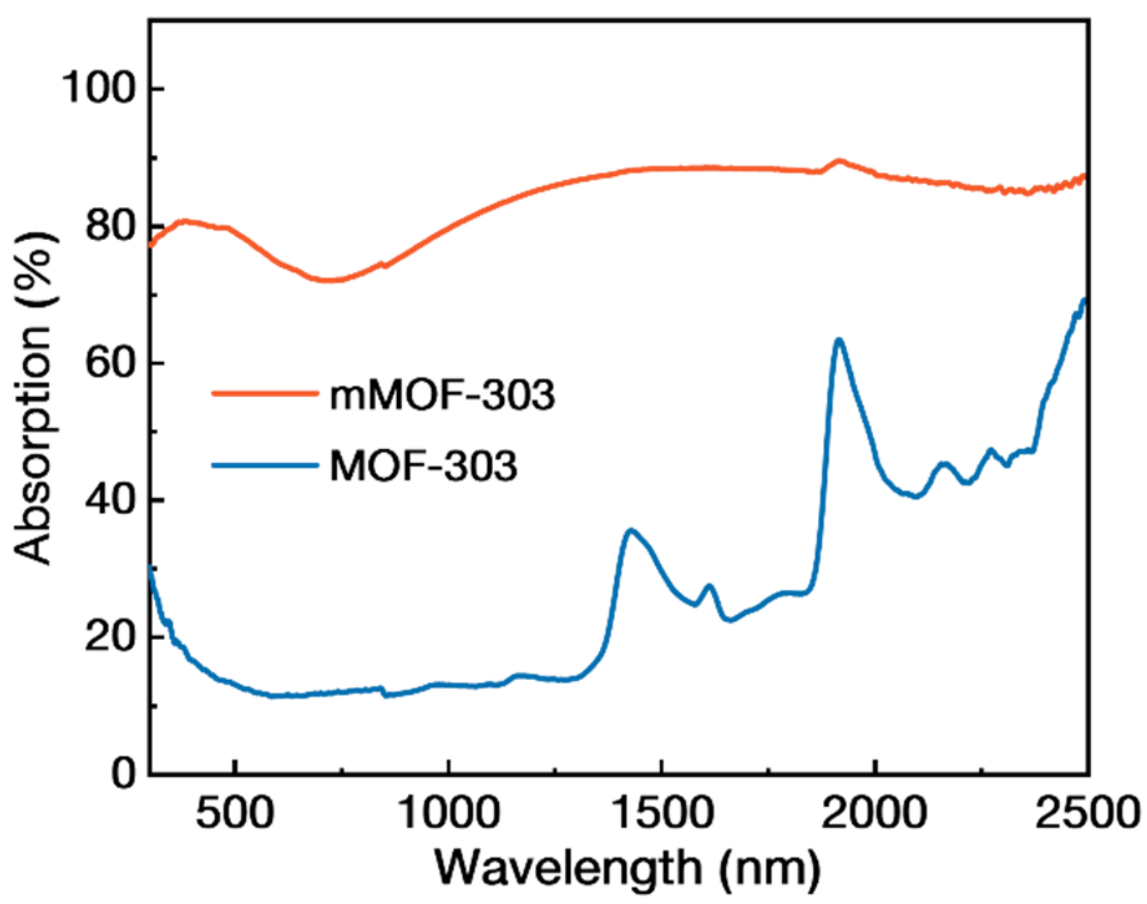

Fig. S26.

UV-vis-NIR absorption spectra of MOF-303 and mMOF-303.

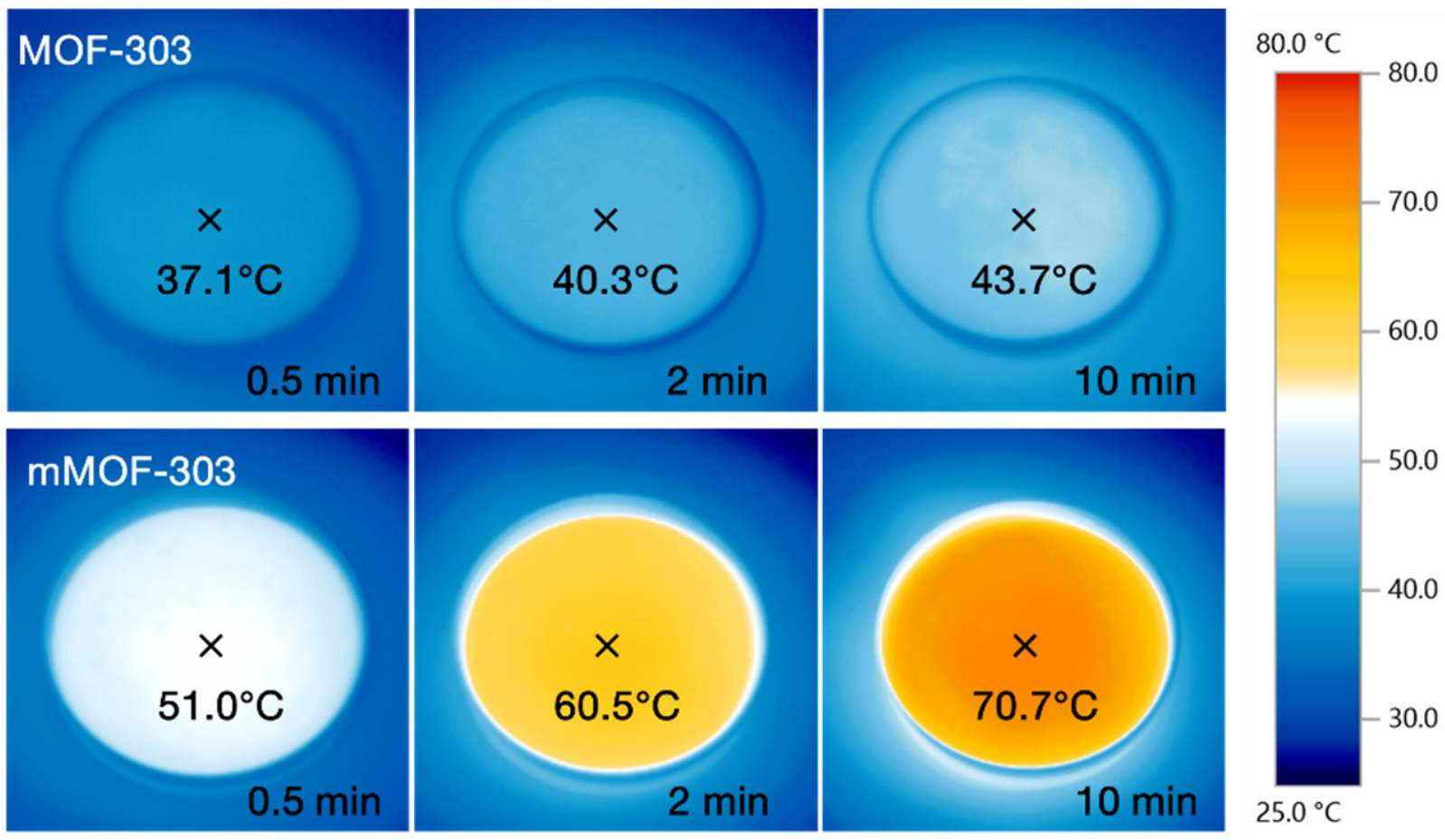

Fig. S27.

Infrared thermal images of photothermal heating measurement for MOF-303 and mMOF-303 at different durations.

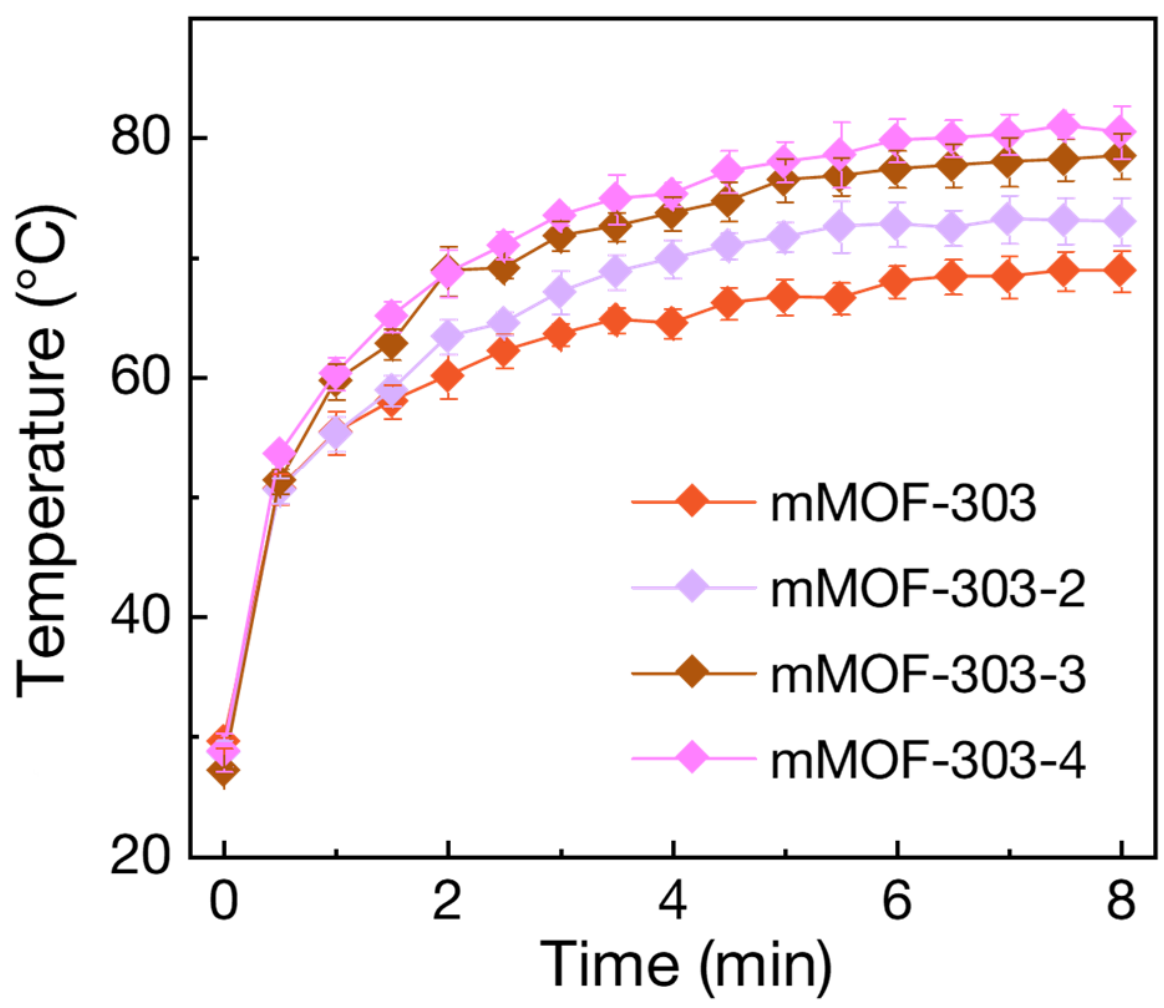

Fig. S28.

Time-dependent photothermal temperature change curves of mMOF-303, mMOF-303-2, mMOF-303-3 and mMOF-303-4.

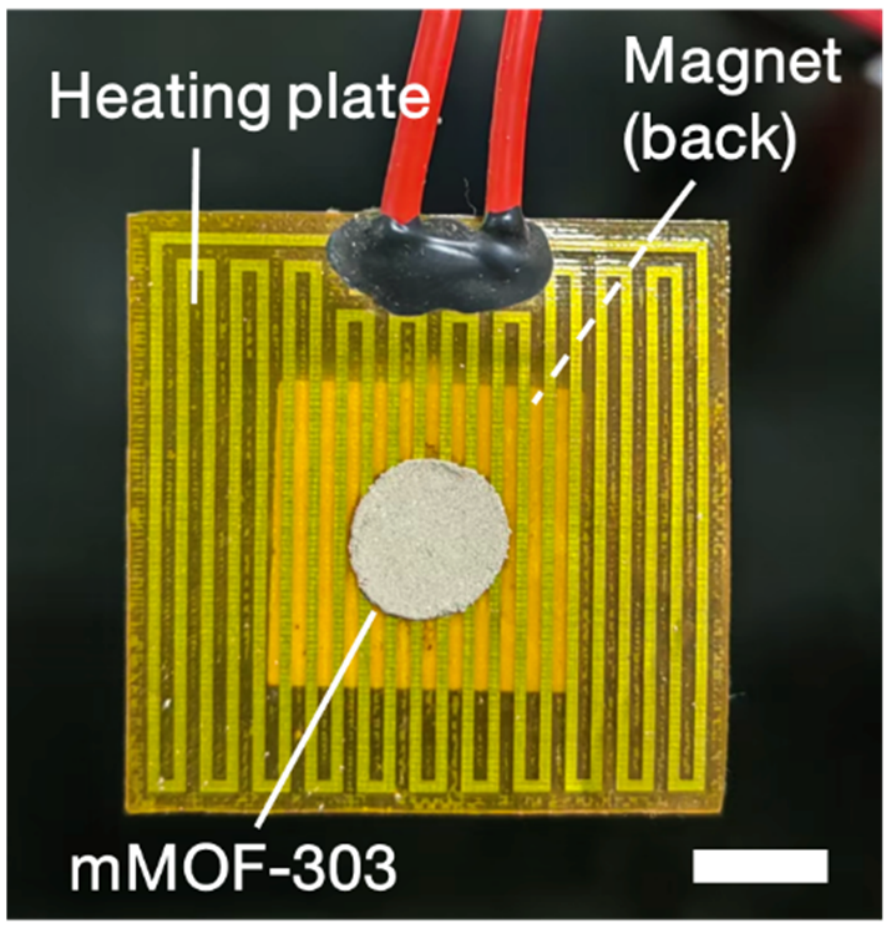

Fig. S29.

Photograph of mMOF-303 magnetically anchored to a suspended electrothermal plate. Scale bar, 1 cm. The applied magnetic field strength is ~ 480.2 mT.

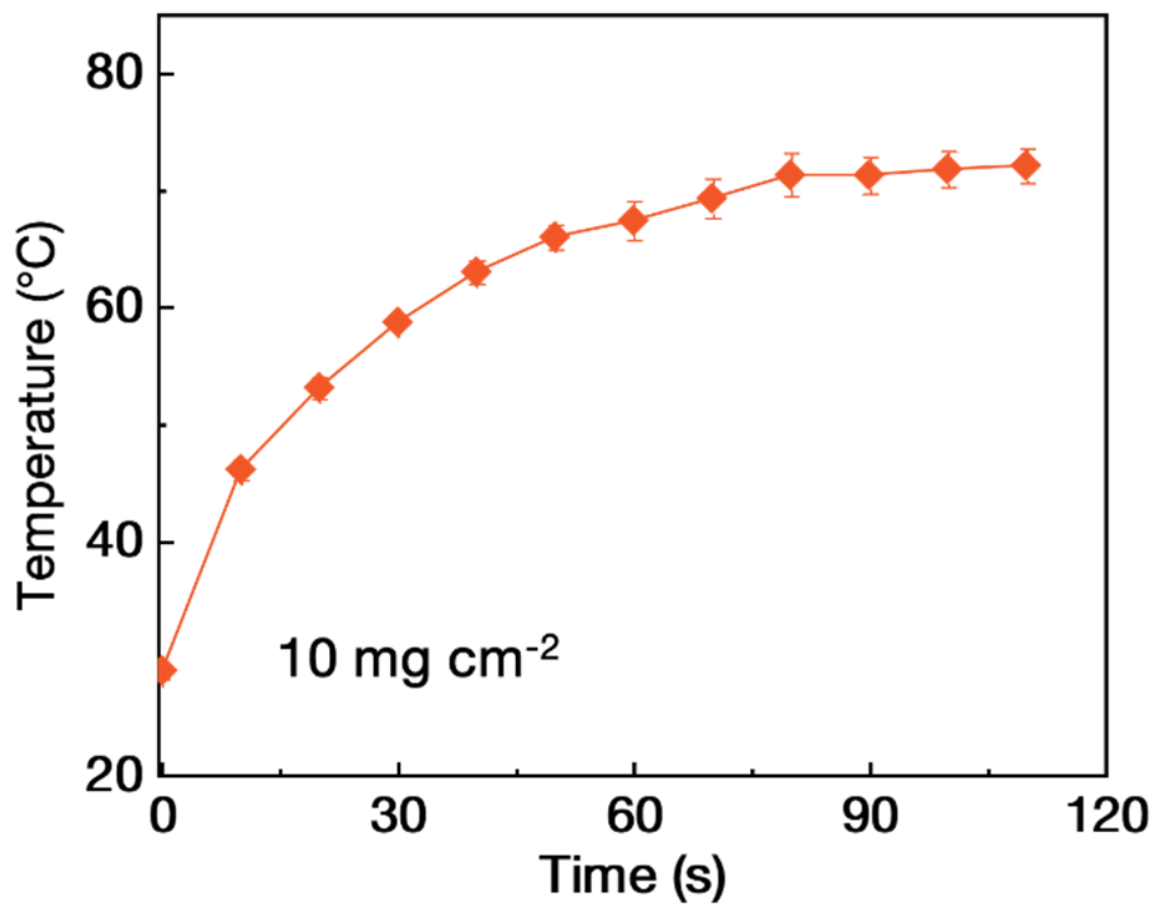

Fig. S30.

Time-dependent temperature change curve of electrothermal heating for mMOF-303. Sample loading density is 10 mg cm^{-2}.

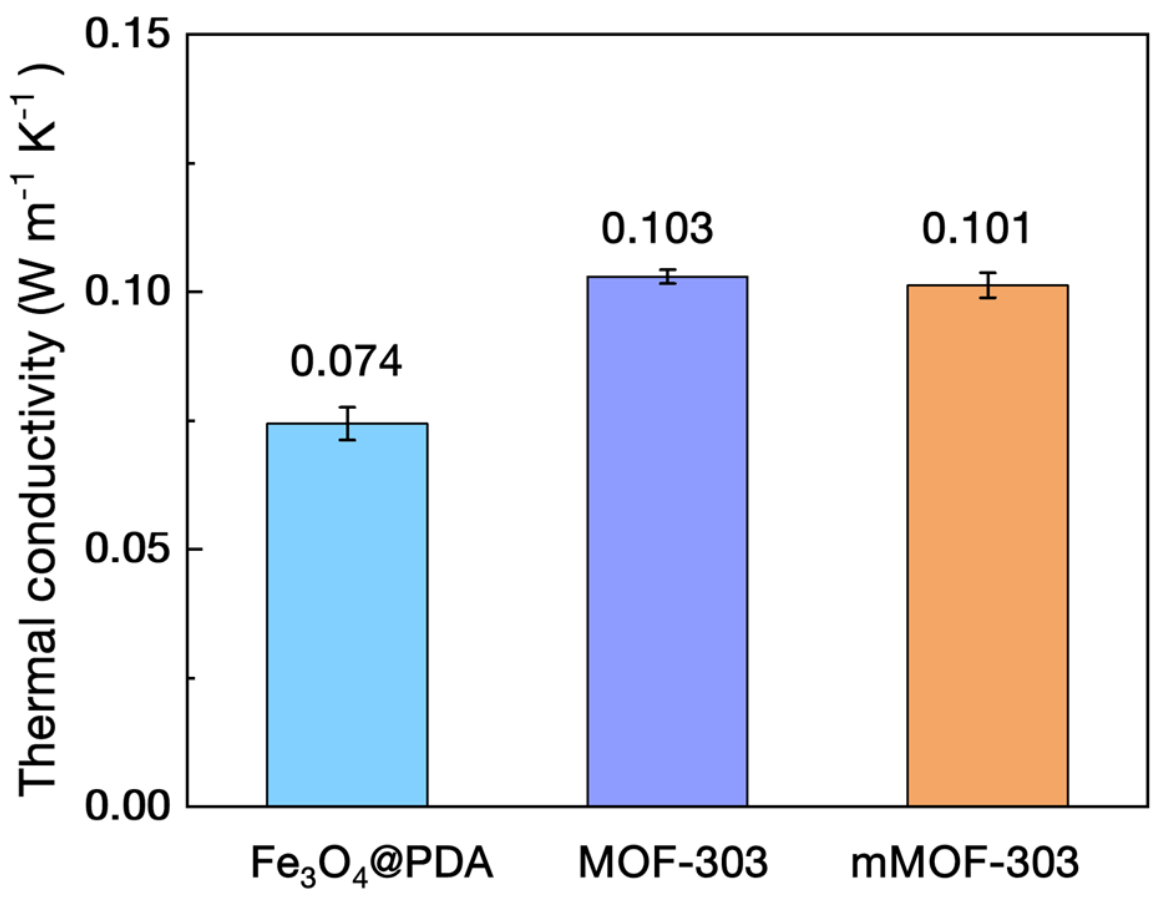

Fig. S31.

Thermal conductivity testing of Fe_3O_4@PDA, MOF-303 and mMOF-303.

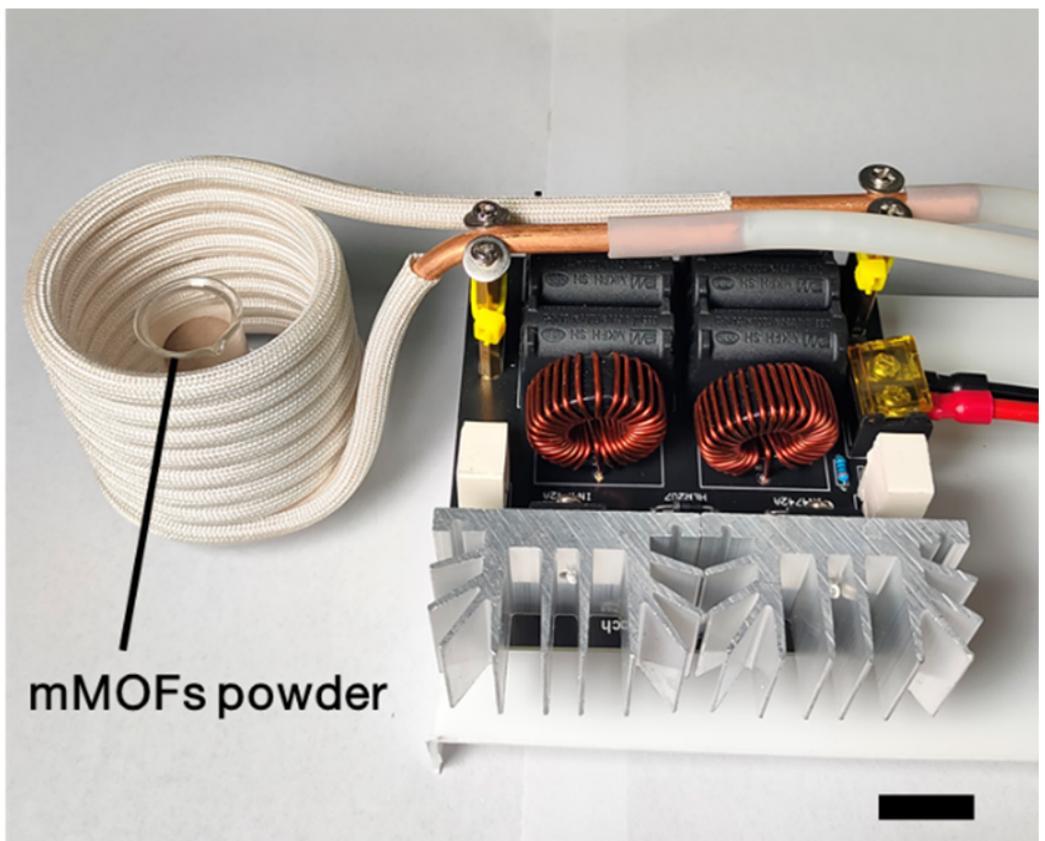

Fig. S32.
Photograph of magnetothermal heating setup. Scale bar, 2 cm.

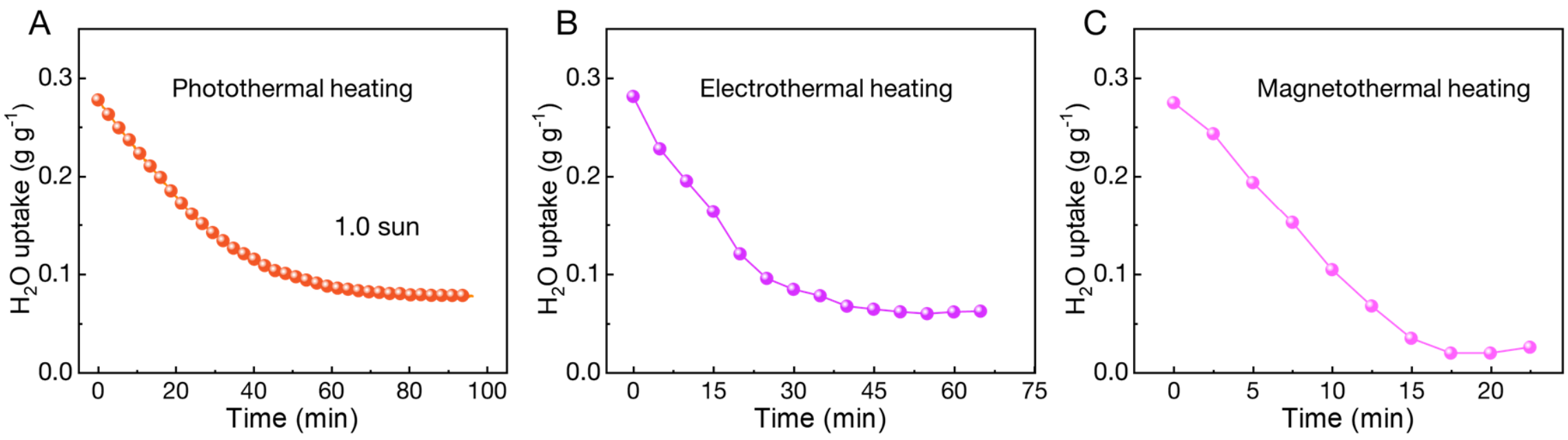

Fig. S33.

Desorption curves of moisture-adsorbed mMOF-303 driven by (A) photothermal, (B) electrothermal and (C) magnetothermal heating.

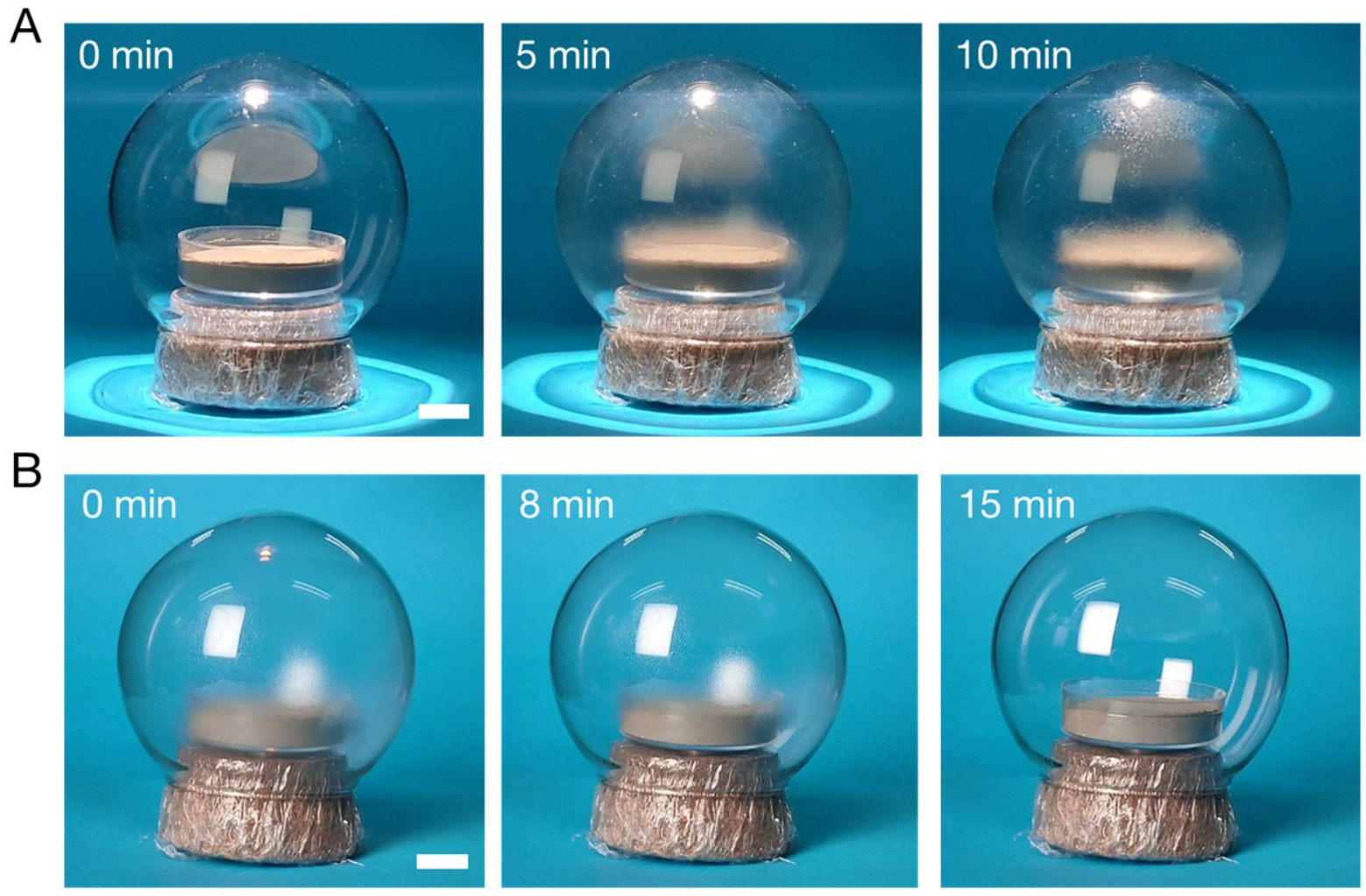

Fig. S34.

Photothermal-driven mMOF-303 (A) water release and (B) water adsorption process. Scale bars, 1 cm.

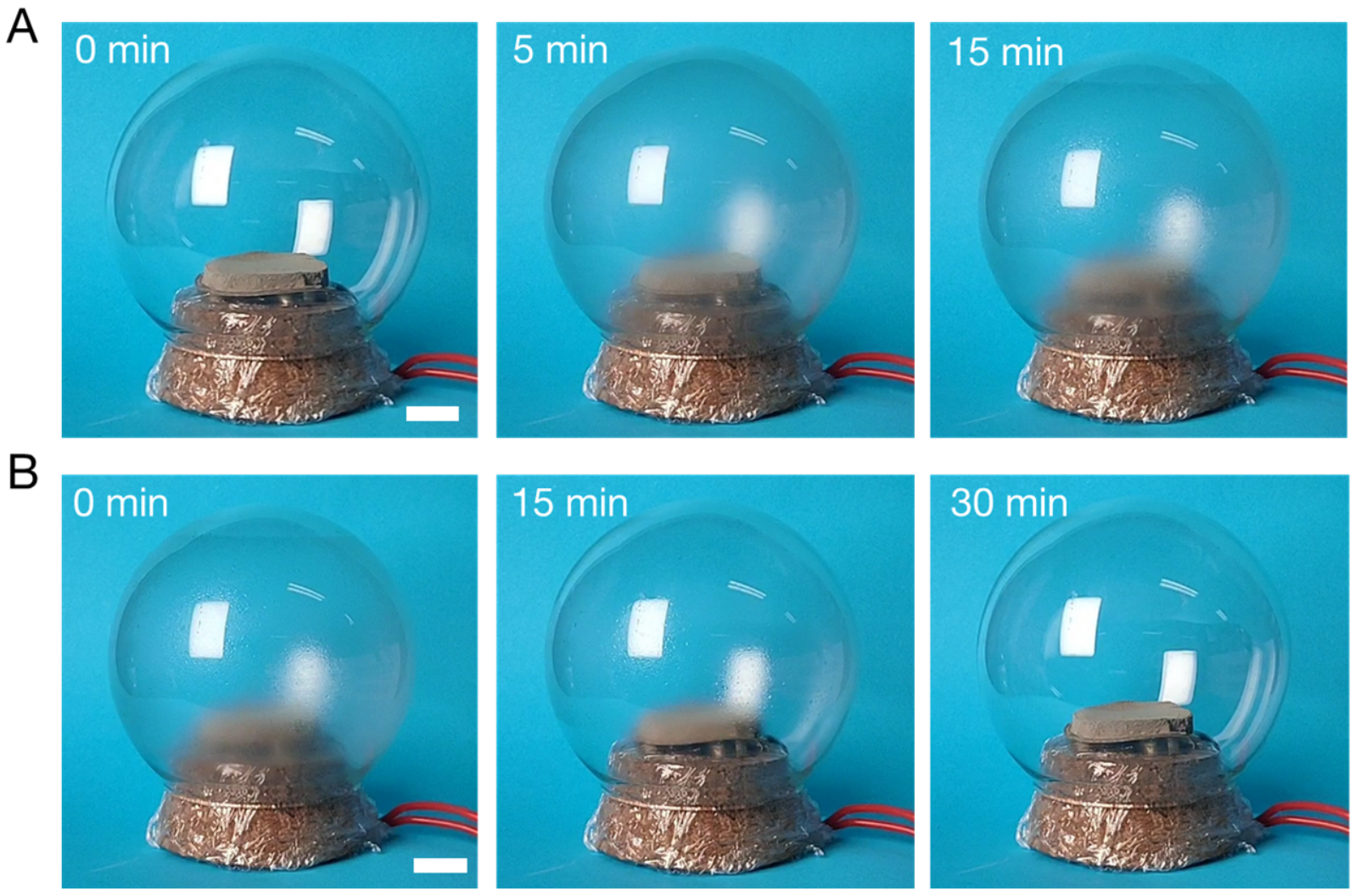

Fig. S35.

Electrothermal-driven mMOF-303 (A) water release and (B) water adsorption process. Scale bars, 1 cm.

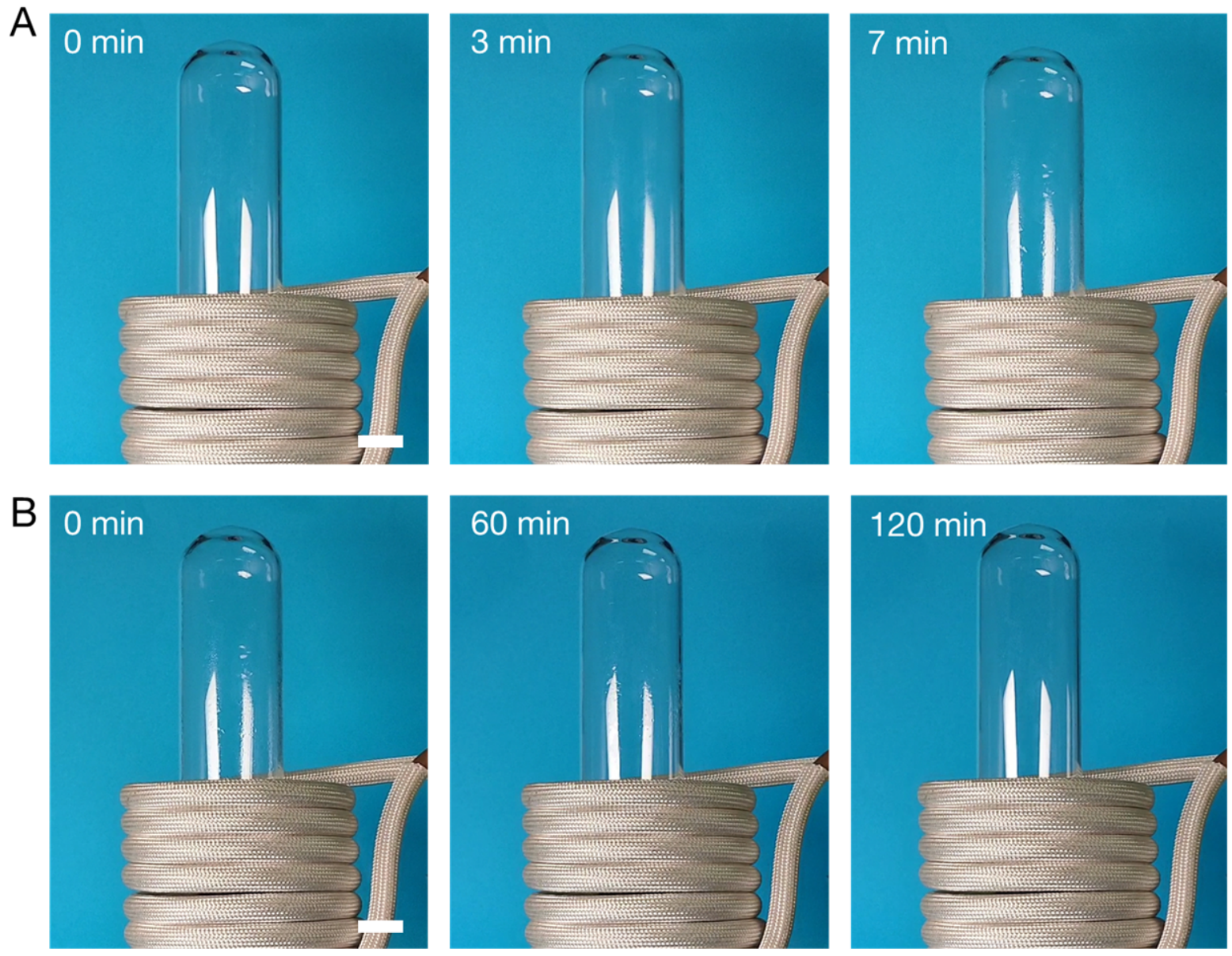

Fig. S36.

Magnetothermal-driven mMOF-303 (A) water release and (B) water adsorption process. Scale bars, 1 cm.

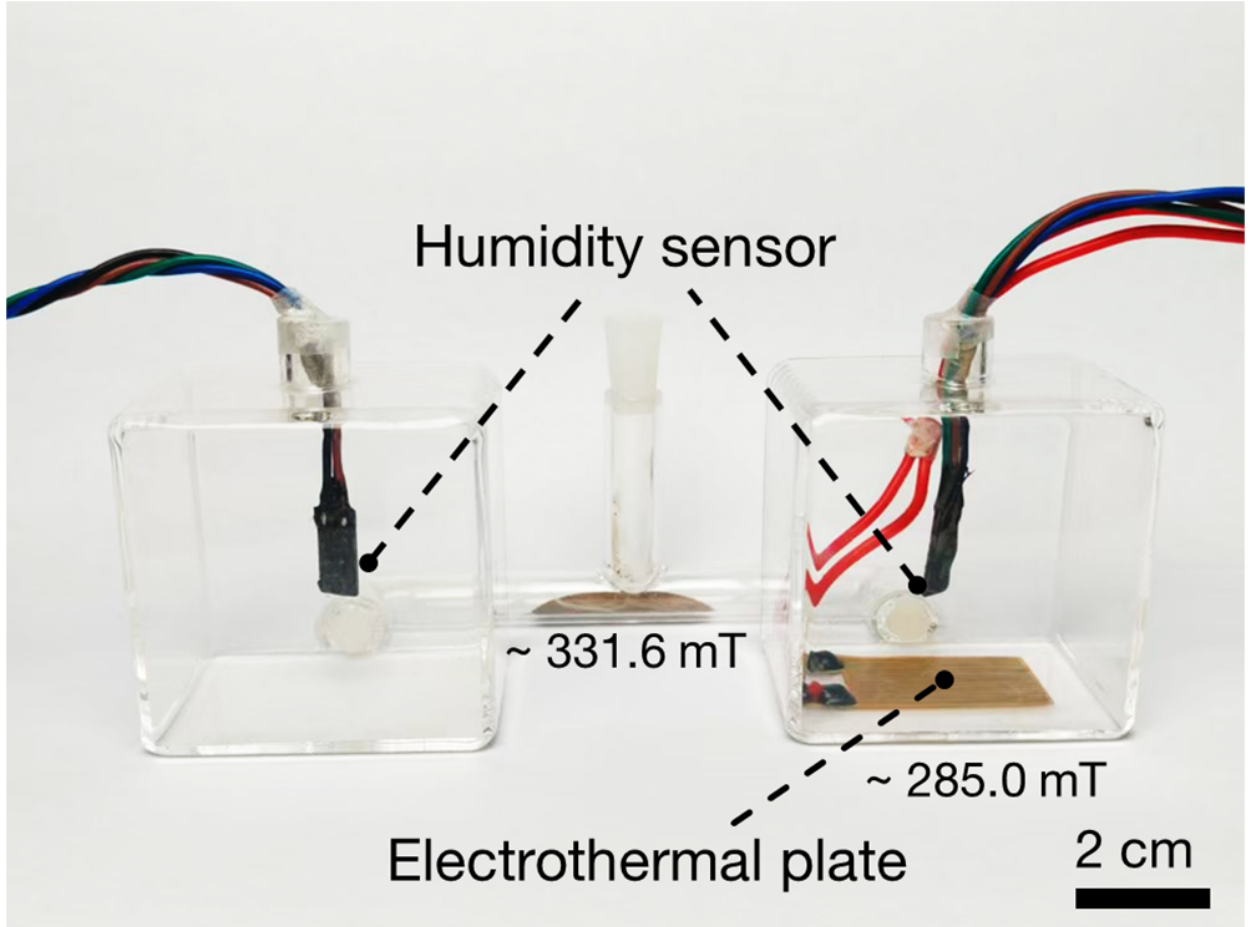

Fig. S37.

Experimental setup for mMOF-303-based humidity regulation.

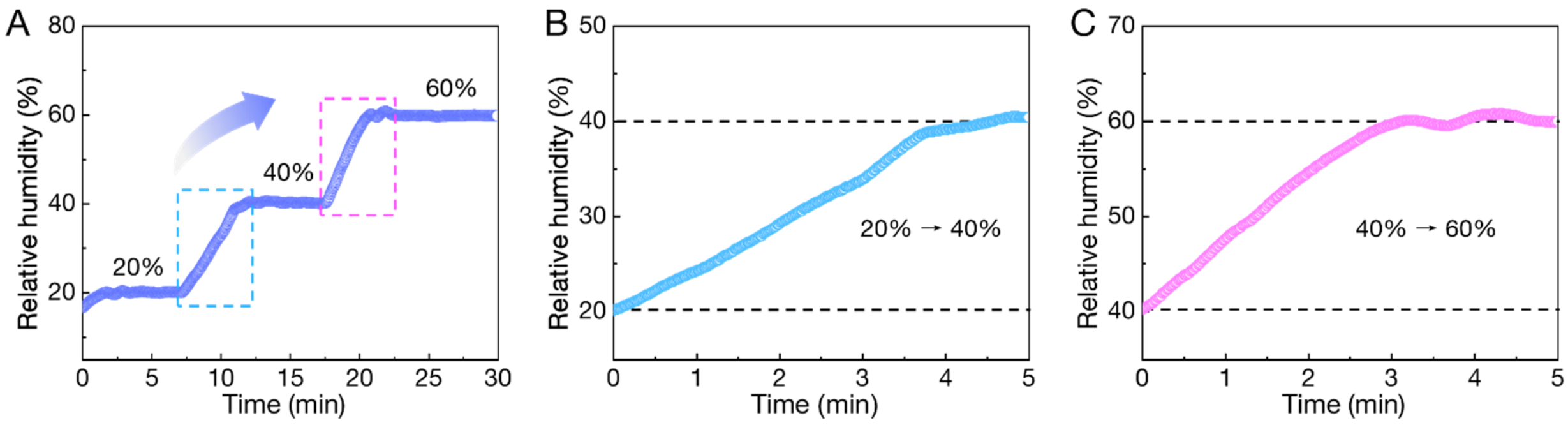

Fig. S38.

Stepwise humidity regulation profiles of mMOF-303. (A) Continuous regulation of different humidity levels. Enlarged curves showing the continuous humidity transition **(B)** from 20% to 40% and **(C)** from 40% to 60%.

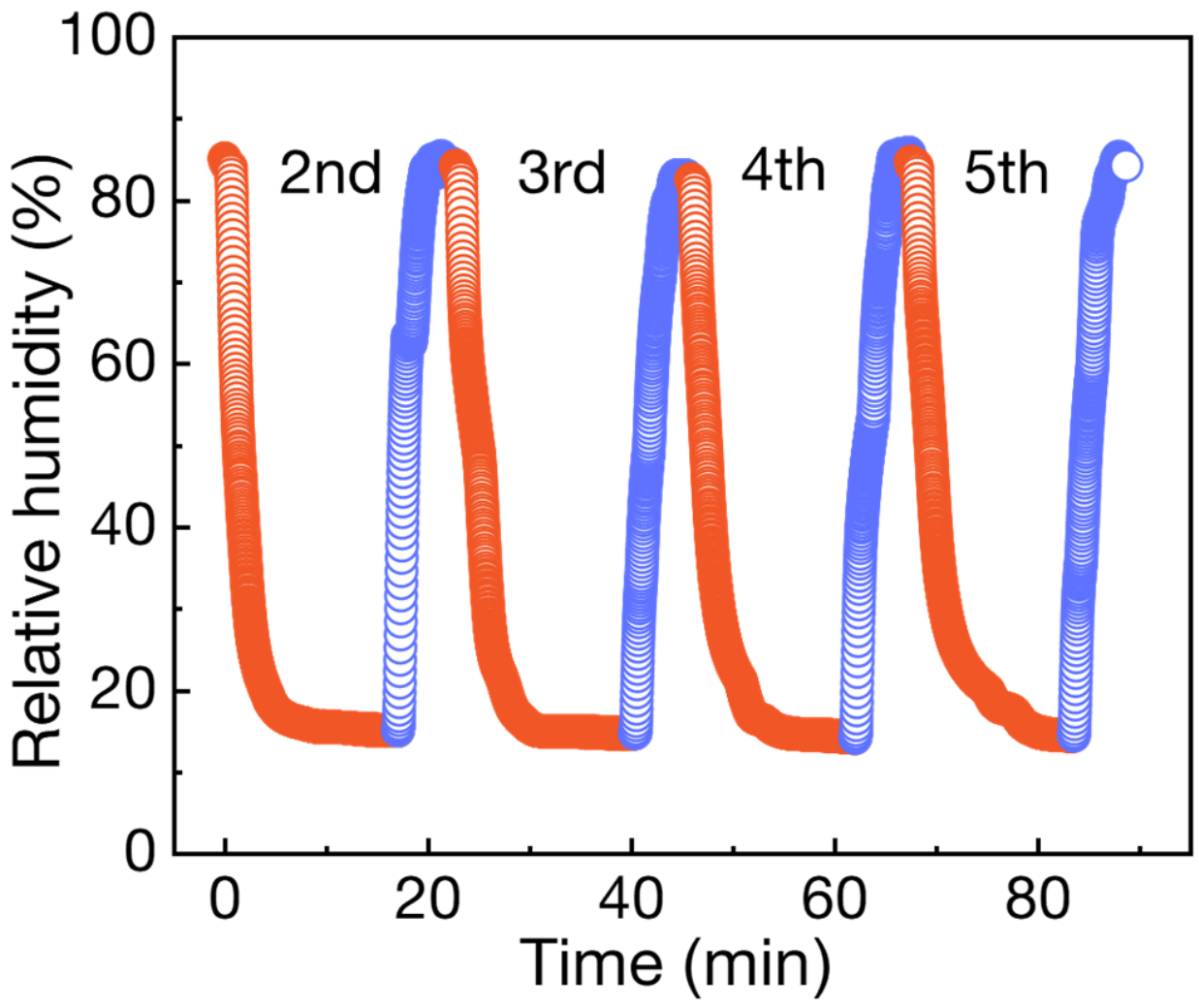

Fig. S39.

Multiple cycle tests of mMOF-303 microbots for humidity regulation.

Table S1.

Comparison of magnetic and water adsorption properties across four magnetic MOF-303 variants.

Samples	Saturation magnetization (emu g^{-1})	Water adsorption isotherms shape	Adsorption inflection point (RH)	Adsorption capacity at 30% RH (cm^3 g^{-1} STP)
mMOF-303	7.1	S-shape	12.5%	397.5
mMOF-303-2	13.8	S-shape	13.0%	371.9
mMOF-303-3	17.3	S-shape	12.3%	336.4
mMOF-303-4	24.6	S-shape	11.6%	281.4

Table S2.

Comparison of water adsorption rates between mMOF-303 and other representative MOF-based sorbents.

**Samples**	**Sample geometry**	**Adsorption conditions**	**Adsorption capacity (g g^{-1})**	**Adsorption time (min)**	**Adsorption Rate (mg g^{-1} min^{-1})**	**Reference**
MOF-801 (1mm)	Packed powder layer	25 °C, 20% RH	~0.25	~120	**~2.1**	(*48*)
MOF-801 (3mm)		25 °C, 20% RH	~0.25	~480	**~0.5**	
MOF-801 (5mm)		25 °C, 20% RH	~0.25	~1200	**~0.2**	
Al-Fum/CNTs/SA	Freeze-dried monolith	25 °C, 35% RH	0.27	210	**~1.3**	(*49*)
CB-coated MOF-303 silk	Thin fiber	25 °C, 25-35% RH	0.28	40	**~7.1**	(*22*)
MIL-101(Cr) nanofiber	Spray-electrospun membrane	25 °C, 50% RH	0.26	80	**~4.3**	(*50*)
Al-Fum/SA	Thin layer	25 °C, 45% RH	0.28	~180	**~1.6**	(*51*)
MOF-303/Chitosan	Freeze-dried aerogel	25 °C, 20% RH	~0.26	20	**~13.0**	(*24*)
		25 °C, 40% RH	~0.3	20	**~15.0**	
MOF-303 pellets	Compressed pellets	25 °C, 40% RH	~0.3	~17	**~17.6**	(*23*)
MIL-101(Cr)/SA	Freeze-dried aerogel	25 °C, 10% RH	~0.13	120	**~1.1**	(*52*)
		25 °C, 30% RH	~0.37	120	**~3.1**	
		25 °C, 40% RH	~0.73	120	**~6.1**	
		25 °C, 50% RH	~1.07	120	**~8.9**	
**mMOF-303**	**Fluidized magnetic powder**	**25 °C, 30% RH**	**~0.32**	**18**	**~17.8**	**This work**

Movie S1.
Magnetic-actuated transition of mMOF-303 microbots from aggregation to dispersion.

Movie S2.
Magnetic-actuated transition of mMOF-303 microbots from dispersion to aggregation.

Movie S3.
The mMOF-303 microbots moving along a customized circular route.

Movie S4.
Directional motion of mMOF-303 microbots through a sealed glass channel.

Movie S5.
Co-rotation of mMOF-303 microbots with the integrated setup.

Movie S6.
Fluidization process of mMOF-303 microbots.

Movie S7.
Photothermal-driven mMOF-303 water release and water adsorption process.

Movie S8.
Electrothermal-driven mMOF-303 water release and water adsorption process.

Movie S9.
Magnetothermal-driven mMOF-303 water release and water adsorption process.
